# MAIT cells are activated during human viral infections

**DOI:** 10.1038/ncomms11653

**Published:** 2016-06-23

**Authors:** Bonnie van Wilgenburg, Iris Scherwitzl, Edward C. Hutchinson, Tianqi Leng, Ayako Kurioka, Corinna Kulicke, Catherine de Lara, Suzanne Cole, Sirijitt Vasanawathana, Wannee Limpitikul, Prida Malasit, Duncan Young, Laura Denney, STOP-HCV consortium, Eleanor Barnes, Jonathan Ball, Gary Burgess, Graham Cooke, John Dillon, Charles Gore, Graham Foster, Neil Guha, Rachel Halford, Cham Herath, Chris Holmes, Anita Howe, Emma Hudson, William Irving, Salim Khakoo, Diana Koletzki, Natasha Martin, Tamyo Mbisa, Jane McKeating, John McLauchlan, Alec Miners, Andrea Murray, Peter Shaw, Peter Simmonds, Chris Spencer, Paul Targett-Adams, Emma Thomson, Peter Vickerman, Nicole Zitzmann, Michael D. Moore, Paolo Fabris, Maria Teresa Giordani, Ye Htun Oo, Stephen M. Laidlaw, Lynn B. Dustin, Ling-Pei Ho, Fiona M. Thompson, Narayan Ramamurthy, Juthathip Mongkolsapaya, Christian B. Willberg, Gavin R. Screaton, Paul Klenerman

**Affiliations:** 1 Peter Medawar Building for Pathogen Research and Translational Gastroenterology Unit, Nuffield Department of Clinical Medicine, University of Oxford, Oxford OX1 3SY, UK; 2 Division of Immunology and Inflammation, Department of Medicine, Faculty of Medicine, Imperial College, Hammersmith Campus, London W12 0NN, UK; 3 Sir William Dunn School of Pathology, University of Oxford, University of Oxford, South Parks Road, Oxford OX1 3RE, UK; 4 MRC Human Immunology Unit, Weatherall Institute of Molecular Medicine, Nuffield Department of Clinical Medicine, University of Oxford, Oxford OX3 9DU, UK; 5 Department of Pediatric, Khon Kaen Hospital, Khon Kaen 40000, Thailand; 6 Department of Pediatric, Songkhla Hospital, Songkhla 90100, Thailand; 7 BIOTEC, NSTDA, Phatumthani 12120, Thailand; 8 Faculty of Medicine, Siriraj Hospital, Mahidol University, Bangkok 10700, Thailand; 9 Nuffield Department of Anesthesia, The John Radcliffe Hospital, Oxford OX3 9DU, UK; 10 STOP-HCV, Stratified Medicine to Optimise the Treatment of Patients with Hepatitis C Virus Infection, Medical Research Council (MRC) Funded Consortium, Oxford OX1 3SY, UK; 11 Infectious Diseases and Tropical Medicine Unit, San Bortolo Hospital, Vicenza, VI 37, Italy; 12 Centre for Liver Research & NIHR Biomedical Research Unit in Liver Disease, University of Birmingham, Birmingham B15 2TT, UK; 13 Nuffield Department of Orthopaedics, Rheumatology, and Musculoskeletal Sciences, Kennedy Institute of Rheumatology, University of Oxford, Oxford OX3 7 LF, UK; 14 Oxford NIHR Biomedical Research Centre, The John Radcliffe Hospital, Oxford OX3 9DU, UK; 15 University of Oxford, Peter Medawar Building for Pathogen Research, South Parks Road, Oxford OX1 3SY, UK; 16 University of Nottingham, Queen's Medical Centre, Nottingham NG7 2UH UK; 17 Conatus Pharmaceuticals, 16745 West Bernardo Drive, Suite 200, San Diego, California 92127, USA; 18 Imperial College London, Wright Fleming Institute, London, UK; 19 University of Dundee, Ninewells Hospital & Medical School, Dundee DD1 9SY, UK; 20 Hepatitis C Trust, 27 Crosby Row, London SE1 3YD, UK; 21 Queen Mary's University of London, 4 Newark Street, London E1 4AT, UK; 22 Gilead Sciences, Stockley Park, 2 Roundwood Avenue, Middlesex UB11 1AF, UK; 23 University of Oxford, 24-29 St Giles', Oxford OX1 3LB, UK; 24 BC Centre for Excellence in HIV/AIDS, St Paul's Hospital, 608-1081 Burrard Street, Vancouver British Columbia, Canada V6Z 1Y6; 25 University of Southampton, University Road, Southampton SO17 1BJ, UK; 26 Janssen Diagnostics, Turnhoutseweg, 30, Beerse 2340, Belgium; 27 UC San Diego, La Jolla, California 92093-0507, USA; 28 Public Health England, 61 Colindale Avenue, London NW9 5EQ, UK; 29 University of Birmingham, Centre for Human Virology, Edgbaston, Birmingham B15 2TT, UK; 30 University of Glasgow, MRC-CVR, 464 Bearsden Road, Glasgow G61 1QH, UK; 31 London School of Hygiene & Tropical Medicine, 15-17 Tavistock Place, London WC1H 9SH, UK; 32 OncImmune Limited, Clinical Sciences Building, Nottingham City Hospital, Hucknall Road, Nottingham NG5 1PB, UK; 33 Merck & Co., Inc., Kenilworth, New Jersey 07033, USA; 34 University of Oxford, Wellcome Trust Centre for Human Genetics, Roosevelt Drive, Oxford OX3 7BN, UK; 35 Medivir AB, Box 1086, Huddinge 141 22, Sweden; 36 University of Bristol, Oakfield House, Oakfield Grove, Clifton BS8 2BN, UK; 37 University of Oxford, South Parks Road, Oxford OX1 3QU, UK

## Abstract

Mucosal-associated invariant T (MAIT) cells are abundant in humans and recognize bacterial ligands. Here, we demonstrate that MAIT cells are also activated during human viral infections *in vivo*. MAIT cells activation was observed during infection with dengue virus, hepatitis C virus and influenza virus. This activation—driving cytokine release and Granzyme B upregulation—is TCR-independent but dependent on IL-18 in synergy with IL-12, IL-15 and/or interferon-α/β. IL-18 levels and MAIT cell activation correlate with disease severity in acute dengue infection. Furthermore, HCV treatment with interferon-α leads to specific MAIT cell activation *in vivo* in parallel with an enhanced therapeutic response. Moreover, TCR-independent activation of MAIT cells leads to a reduction of HCV replication *in vitro* mediated by IFN-γ. Together these data demonstrate MAIT cells are activated following viral infections, and suggest a potential role in both host defence and immunopathology.

## Abstract (web summary)

Mucosal Associated Invariant T cells have been implicated in response to bacterial pathogens. Here the authors show that in human viral infections, these cells are activated by IL-18 in cooperation with other pro-inflammatory cytokines, producing interferon gamma and granzyme B.

## Graphical abstract

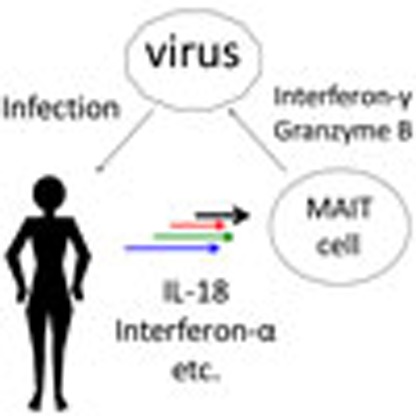

Mucosal-associated invariant T (MAIT) cells are an abundant and distinctive T cell subset first identified by Porcelli *et al*.^1^ in 1993 in humans on the basis of the invariant V-alpha chain (Vα7.2-Jα33) and later described in mice^2^. MAIT cells comprise ∼5–10% of T-cells within the periphery and are found enriched in both the gut and in the liver—where in humans they may represent between ∼12% and 50% of T-cells^3^,^4^,^5^,^6^. In both mouse and man, MAIT cells are restricted to a bacterial riboflavin synthesis pathway-derived ligand presented by the conserved MHC-class I like molecule MR1 (refs 7, 8). *In vivo* challenge models using riboflavin synthesizing bacteria, such as *Klebsiella* species and mycobacteria, have confirmed an anti-bacterial role for MAIT cells^9^,^10^. MR1 tetramers loaded with the ligand rRL-6-CH_2_OH have allowed the specific detection of human and murine MAIT cells^11^.

In recent years, a number of groups have elucidated the distinct phenotypic and functional profile of MAIT cells, characterized by the high expression of the C-type lectin CD161 (KLRB1 or NKR-P1A), and the capacity to secrete multiple cytokines, including IL-17, interferon (IFN)-γ and TNF-α^12^,^13^,^14^,^15^,^16^. The distinctive phenotype of MAIT cells appears to be driven by two key transcription factors, RORγt and PLZF^3^,^6^,^13^,^17^. RORγt expression is linked to development of type 17 function and expression of surface receptors such as IL23R and CCR6 (refs 5, 18). This is consistent with mucosal defence and anti-bacterial functions and also consistent with the bacterial specificity of the receptor. PLZF is critical for development of invariant natural killer T cell (iNKT) cells and may be responsible for a distinct set of ‘innate' phenotypic features, including marked upregulation of the pro-inflammatory cytokine receptors IL-18R and IL-12R^19^,^20^. This dual transcriptional drive suggests that MAIT cells may possess multiple parallel functionalities or modes of activation.

Given the specificity of the T-cell receptor (TCR), it appears that activation of MAIT cells *in vivo* is driven by responsiveness to bacteria (and some yeasts)^21^. However, given their ‘innate' phenotype, broad range of effector functions, and tissue distribution, we addressed the question of whether they may also have evolved to respond to viral infections *in vivo*. If so, this would markedly widen the role of MAIT cells in human infection.

Here, we addressed MAIT cell activation in response to a set of major human viral pathogens both *in vivo* and *in vitro*. *In vivo*, MAIT cells are activated in response to dengue virus (DENV), hepatitis C virus (HCV) and influenza virus. *In vitro*, DENV, HCV and influenza virus readily trigger MAIT cell populations in an IL-18 dependent manner, in concert with other virally-driven pro-inflammatory cytokines, including IFN-α/β. Activation of MAIT cells in acute dengue correlated both with disease outcome and with IL-18 signals. Similarly, *in vivo* activation of MAIT cells during HCV therapy correlated with specific addition of IFN-α during therapy. Taken together, these data strongly implicate a role for MAIT cells in response to major virus infections of man and provide a mechanism for their virus-responsive nature. Overall, this significantly expands the pathogen response repertoire of this abundant human T-cell subset.

## Results

### MAIT cell activation during acute viral infections *in vivo*

**Figure 1:**
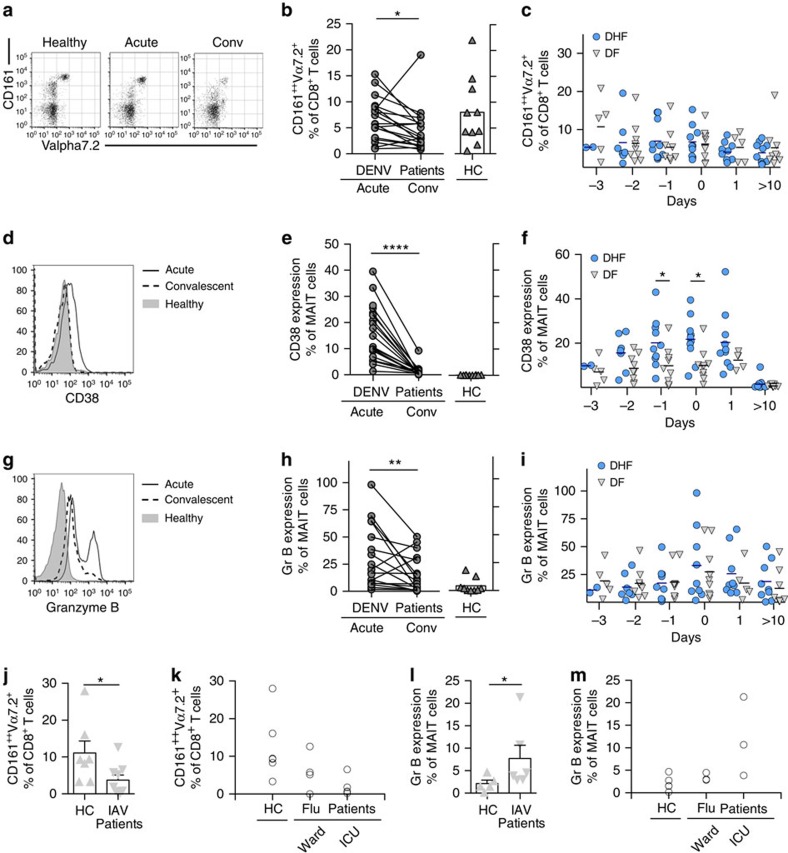
MAIT cell activation during acute viral infections *in vivo.* PBMC's from healthy controls (HC, *n*=5–10), patients suffering from severe dengue (DHF, *n*=2–10), dengue (DF, *n*=4–10) or acute, hospitalized patients infected with influenza A virus (*n*=7–9) were analysed by flow cytometry by gating on live CD3^+^CD8^+^CD161^++^Vα7.2^+^ (MAIT) cells. (**a**,**d**,**g**) Representative flow cytometry plots. (**b**,**e**,**h**) Comparison between acute (day 0) and convalescent phase (day >10) of infection. (**c**,**f**,**i**) Comparison between DHF and DF patients. (**j**–**m**) Acute, hospitalized patients infected with influenza virus. (**b**,**c**,**j**,**k**) MAIT cell frequency as a proportion of the CD8^+^ T cell population. (**e**,**f**) Percentage of CD38 expression by MAIT cells. (**h**,**i**,**l**,**m**) Percentage of Granzyme B expression by MAIT cells. Statistical significance was determined with a Wilcoxon matched-paired test (**b**,**e**,**h**) or Mann–Whitney test (**j**,**k**). Bars represent means±s.e.m. ns>0.05, *P 0.05, **P≤0.01, ***P≤0.001, ****P≤0.0001. conv, convalescent; Gr B, Granzyme B; HC, healthy control; ICU, intensive care unit.

Whether human MAIT cells respond during acute viral infections remains unclear. To address this we analysed MAIT cell activation in DENV and influenza virus infection. In this study, MAIT cells were defined as CD161^++^Vα7.2^+^ CD8^+^CD4^−^CD3^+^ T cells. DENV infection presents with a rapid onset of a febrile illness, which lasts around a week. After the virus has been cleared and fever drops, patients follow two distinct courses: an uncomplicated resolution (dengue fever; DF) or a severe course associated with organ dysfunction and haemorrhage (dengue haemorrhagic fever; DHF). The determinants driving outcome are still poorly understood. However, an aberrant immune response, around the day of defervescence, is thought to have an important role in the pathogenesis. We analysed MAIT cells in a longitudinal study of peripheral blood mononuclear cells (PBMC) from a cohort of patients with DHF or DF. Blood samples were collected at different time points during the acute phase of infection and dates were named in relation to the day fever subsided (day of defervescence, day 0). Samples taken 10 or more days after the day of defervescence (day >10) were considered convalescent. We found a small but significant reduction in MAIT cell frequencies between the acute phase (day 0) and the convalescent phase (day >10) of infection (Fig. 1a,b), but no differences in MAIT cell frequency between DHF and DF patients (Fig. 1c).

Analysis of CD38 revealed marked *in vivo* activation of MAIT cells (Fig. 1d,e), which increased over the course of infection and peaked at a critical moment for DENV infected patients—the day of defervescence. Interestingly, patients who developed the severe form of dengue had higher levels of MAIT cell activation as judged by CD38 expression compared to DF patients over the course of acute infection (Fig. 1f). MAIT cell activation resolved to healthy control levels in the convalescent sample (Fig. 1d,e).

Granzyme B expression was also assessed due to its tight regulation in MAIT cells, and its absence in cells from healthy donors^3^,^22^. Furthermore, upregulation of Granzyme B is associated with the acquisition of cytolytic function by MAIT cells^22^,^23^. We therefore analysed Granzyme B function in acute dengue and found this followed the same time course as that of CD38 (Fig. 1g–i).

Given their role in mucosal defence, we next addressed the activation of MAIT cells in response to influenza virus, a virus with a segmented genome of negative-sense RNA. Again, patients with acute, severe influenza virus infection had reduced MAIT cell frequencies and an increase in Granzyme B expression on MAIT cells (Fig. 1j–m).

Taken together, our results indicate substantial triggering of MAIT cells *in vivo* during acute viral infection.

### MAIT cell activation during chronic viral infection *in vivo*

**Figure 2:**
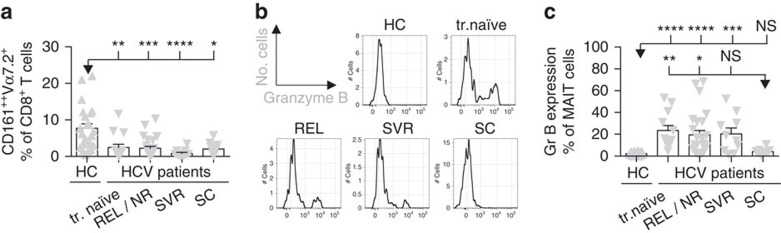
MAIT cell activation during chronic viral infection *in vivo.* PBMC's from healthy controls (*n*=20–23) or patients (*n*=12–25) with persistent (treatment naive, REL, NR) and resolved HCV infection (SVR, SC) were analysed by flow cytometry by gating on live CD3^+^CD8^+^CD161^++^Vα7.2^+^ (MAIT) cells. (**a**) MAIT cell frequency as a proportion of the CD8^+^ T cells. (**b**,**c**) Granzyme B expression by MAIT cells. (**b**) Representative flow cytometry plots. Bars represent means±s.e.m. Statistical significance was determined with the Kruskal–Wallis test followed by the Dunns' test. ns>0.05, *P 0.05, **P≤0.01, ***P≤0.001, ****P≤0.0001. HC, healthy control; Gr B, Granzyme B; REL/NR, relapse/non-response; SC, spontaneous clearance; SVR, sustained virological response; tr., treatment.

MAIT cells are found at high frequencies within the liver in both healthy and diseased states^3^,^5^,^13^,^24^. Therefore, we analysed MAIT cell activation during chronic infection with HCV, like DENV, a member of the *Flaviviridae* family of positive-sense RNA viruses. We examined MAIT cell frequency and phenotype in the PBMC of patients with persistent and resolved HCV infection (spontaneously or after therapy). In all HCV patients, regardless of status, we observed a reduction in MAIT cell frequencies compared to healthy controls (Fig. 2a). However, we only observed upregulation of Granzyme B in patients with prolonged HCV infection (including those who had subsequently responded to antiviral therapy; Fig. 2b,c), and not in those patients with prior short-lived viremia at a distant time-point associated with acute resolving infection (thus, more akin to convalescent DENV infection). Our results indicate substantial activation of MAIT cells *in vivo* both during acute and chronic viral infections.

### Viral MAIT cell activation *in vitro*

Having observed MAIT cell activation *in vivo* during acute and chronic viral infections, we next established *in vitro* models for viral infections using PBMCs or human CD8^+^ T cells, co-incubated with infected or virus-treated dendritic cells (DCs) or macrophages as antigen presenting cells (APC).

**Figure 3:**
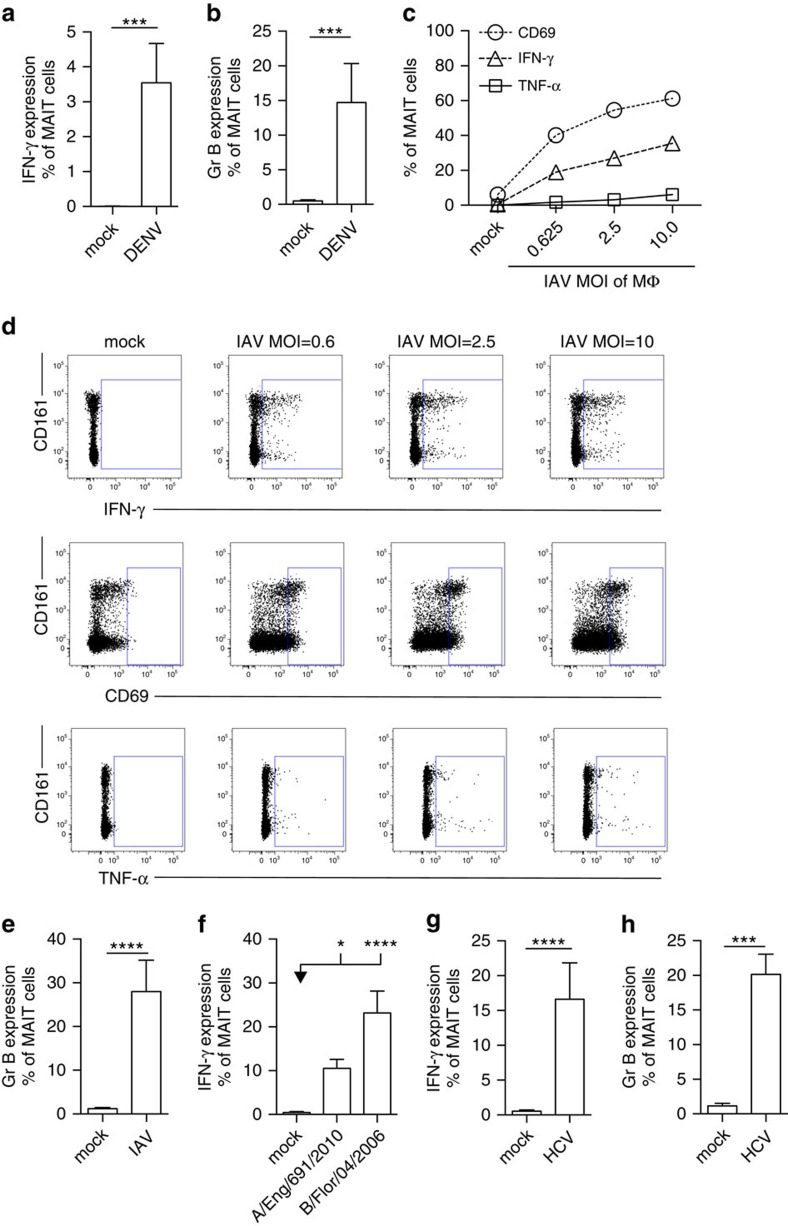
Viral MAIT cell activation *in vitro*. MAIT cells from healthy individuals were analysed by flow cytometry, gated on live CD3^+^CD161^++^Vα7.2^+^ cells. (**a**,**b**) PBMC's (*n*=7) were co-cultured with autologous monocyte-derived DC's exposed to DENV (MOI=1) as described in ‘Methods'. (**c**–**e**) CD8^+^ T cells isolated from PBMC's (*n*=11–12) were co-cultured with IAV-exposed macrophages (MOI=1) as described in ‘Methods', unless indicated otherwise. (**f**) CD8^+^ T cells isolated from PBMC's (*n*=11–12) were co-cultured with macrophages exposed to the clinical H3N2 influenza A strain (A/England/691/2010 (*n*=7)) or influenza B (B/Florida/04/2006 (*n*=8)) (MOI=1) as described in ‘Methods'. (**g**,**h**) CD8^+^ T cells isolated from PBMC's (*n*=7–12) co-cultured with macrophages exposed to HCV (MOI=1) as described in ‘Methods'. Proportion of MAIT cells producing IFN-γ (**a**,**c**,**d**,**f**,**g**), TNF-α (**c**,**d**), CD69 (**c**,**d**) or Granzyme B (**b**,**e**,**h**). (**d**) Representative flow cytometry plots. All data are representative from at least two independent experiments. Bars represent means±s.e.m. Statistical significance was determined with the Kruskal–Wallis test followed by the Dunns' test (**f**) or the Mann–Whitney test (**a**,**b**,**e**,**g**,**h**). ns>0.05, *P 0.05, ***P≤0.001, ****P≤0.0001. Eng, England; Flor, Florida; HC, healthy control; Gr B, Granzyme B; MΦ, macrophage; REL/NR, relapse/non-response; SC, spontaneous clearance; SVR, sustained virological response; tr., treatment.

MAIT cells were readily and specifically activated in response to DENV-treated APCs (multiplicity of infection (MOI)=1), as indicated by production of IFN-γ and Granzyme B (Fig. 3a,b), as well as, expression of CD38, with minimal production of TNF-α (Supplementary Fig. 1a–c). Activation was dependent on the presence of APCs as DENV alone did not activate MAIT cells (data not shown).

We next examined activation of MAIT cells *in vitro* in response to influenza virus. APCs incubated with influenza virus (MOI=1) promoted strong MAIT cell activation, inducing upregulation of CD69 and IFN-γ (with minimal TNF-α secretion), in a dose-dependent manner (Fig. 3c,d). Also, Granzyme B expression was induced (Fig. 3e). This activation was not restricted to laboratory-adapted influenza virus strains, but also was seen in response to recently-circulating strains of influenza A and B (Fig. 3f). Interestingly, MAIT cell activation was also readily induced using PBMCs instead of purified APCs (Supplementary Fig. 1d). Moreover, influenza virus exposure of purified monocyte, macrophage and DC populations all led to MAIT cell activation (Supplementary Fig. 1e).

Finally, we tested the impact of HCV on MAIT cells *in vitro*. MAIT cells expressed IFN-γ and Granzyme B (Fig. 3g,h), as well as CD69 in response APC's treated with HCV (MOI=1), in a dose-dependent and specific manner (Supplementary Fig. 1f–i).

Taken together, we demonstrate that MAIT cells are activated *in vitro* in response to DENV, influenza virus and HCV-exposed APCs.

### Viral MAIT cell activation is dependent on cytokines

**Figure 4:**
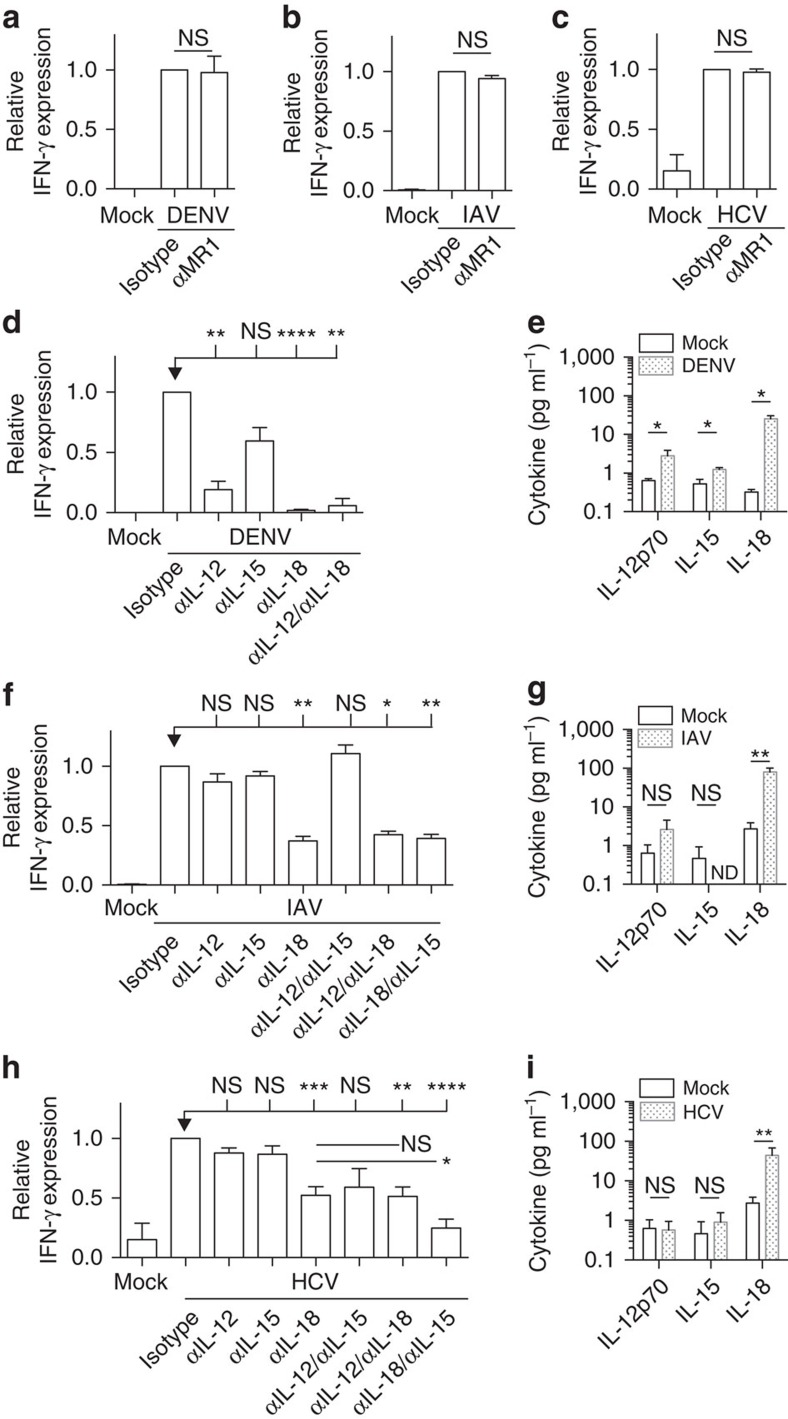
Viral MAIT cell activation is dependent on cytokines. (**a**–**d**,**f**,**h**) Isotype control, anti-MR1, anti-IL-12, anti-IL-15 and/or anti-IL-18 antibodies were added to a co-culture exposed to DENV (**a**,**d**, *n*=3–6), IAV (**b**,**f**, *n*=5–29) or HCV (**c**,**h**, *n*=7–16). IFN-γ expression by MAIT cells (gated on live CD3^+^CD8^+^CD161^++^Vα7.2^+^ cells) was analysed by flow cytometry and is shown relative to the isotype control. (**e**,**g**,**i**) IL-12p70, IL-15 and IL-18 levels secreted by virus-exposed APC's. (**e**) DENV-exposed DC's (*n*=4) at 42 h. (**g**) IAV-exposed macrophages (*n*=7) at 48 h. (**i**) HCV-exposed macrophages (*n*=7) at 48 h. Data are representative from at least two independent experiments. Bars represent means±s.e.m. Statistical significance was determined with the Mann–Whitney test (**a**–**c**,**e**,**g**,**i**) or the Kruskal–Wallis test followed by the Dunns' test or the Mann–Whitney test (**d**,**f**,**h**). ns>0.05, *P 0.05, ***P≤0.001, ****P≤0.0001. ND, not detected.

Next, we investigated the mechanisms of MAIT cells activation in response to viruses. The ligand presented by MR1 is derived from the riboflavin synthesis pathway, which is not found in host cells or viruses^7^. Consistent with this, using the models described above, we confirmed by antibody blocking of MR1 that MAIT cell activation was TCR-independent for all the viruses tested (Fig. 4a–c).

As activation was TCR-independent, we explored triggering of MAIT cells by cytokines. Previously, we have shown that TLR8 is capable of inducing IFN-γ expression in MAIT cells via IL-12 and IL-18 (ref. 24). In addition, IL-15 can specifically activate distinct functions of MAIT cells in synergy with IL-12 and/or IL-18, in a dose-dependent manner (Supplementary Figs 2 and 3)^25^. We extended this finding by exploring responses to a range of TLR ligands in PBMCs and found endosomal TLR3 was also a potent activator (Supplementary Fig. 4). As with TLR8, TLR3 induced MAIT cell activation via IL-18 and IL-12 and not MR1^12^,^13^. TLR sensing by APC's can occur in the absence of viral replication^26^,^27^. To assess the requirement of viral replication for MAIT cell activation, we used ultraviolet-irradiation of the viruses, which prevents transcription or replication. Ultraviolet-irradiated DENV was no longer able to activate MAIT cells (Supplementary Fig. 5a). In contrast, ultraviolet-irradiated HCV and influenza virus were still able to activate MAIT cells, although less efficiently compared to untreated virus (Supplementary Fig. 5b,c). Accordingly, DENV productively infects APCs, whereas productive influenza virus and HCV infection is limited in both primary and stem cell-derived human APCs (Supplementary Fig. 5d–f)^28^,^29^,^30^. Furthermore, the level of DENV infection correlated with MAIT cell IFN-γ expression (Supplementary Fig. 5e).

We next assessed blockade of IL-12, IL-18 and IL-15 in the *in vitro* virus models. Figure 4d reveals that IL-12 and IL-18 had the most significant impact on DENV-dependent MAIT cell activation using DC's, with anti-IL-18 blockade alone leading to complete suppression of IFN-γ expression. IL-18 was the most abundant cytokine induced in DC's in response to DENV (Fig. 4e).

Anti-IL-18 also inhibited activation of MAIT cells induced by influenza virus, a result observed using purified APCs (Fig. 4f) or PBMC's (Supplementary Fig. 6a). Blockade of IL-12 and IL-15 alone or in combination had a limited impact in influenza virus-induced MAIT cell activation using macrophages (Fig. 4f). Accordingly, IL-18, but not IL-12 or IL-15 was secreted by macrophages after incubation with influenza virus (Fig. 4g).

Similar to DENV and influenza virus, IL-18 had a dominant role in HCV-induced MAIT cell activation (Fig. 4h). Addition of further blockade with anti-IL-15 was required to achieve maximal suppression. We assessed expression of IL-12, IL-15 and IL-18 on macrophages after incubation with HCV (Fig. 4i). No IL-15 protein was detected, yet IL-15R (responsible for IL-15 trans-presentation^31^) was upregulated by macrophages in response to HCV, as measured by quantitative PCR (Supplementary Fig. 6b).

Overall, these experiments indicate a mechanism by which viruses can activate MAIT cells, dependent on viral sensing and cytokines, with a key role for IL-18, and independent of MR1 and TCR.

### Virally-triggered IL-18 correlates with MAIT cell activation

**Figure 5:**
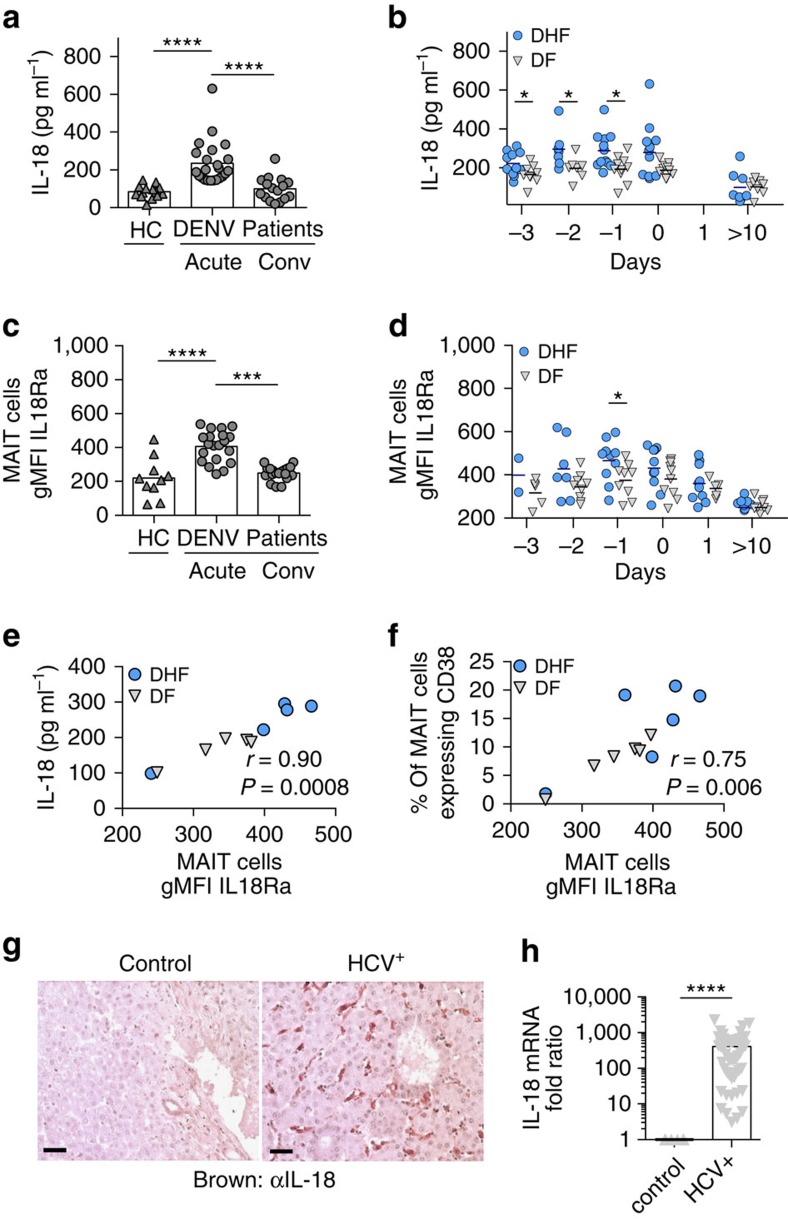
Virally triggered IL-18 correlates with MAIT cell activation. (**a**–**d**) PBMC's from healthy controls (*n*=10) or patients suffering from severe dengue (DHF, *n*=2–12) or dengue (DF, *n*=4–12) were analysed. (**a**,**b**) Plasma levels of IL-18. (**c**,**d**) IL-18Ra expression on MAIT cells. (**a**,**c**) Comparison between acute (day 0) and convalescent (conv) phase (day >10) of infection. (**b**,**d**) Comparison between DHF and DF patients. Correlation of plasma levels of IL-18 (**e**) or MAIT cell CD38 expression (**f**) against MAIT cell IL-18Ra expression by the Spearman-rank correlation test. (**g**) Paraffin-embedded human liver tissues sections from HCV or control patients were stained for IL-18 (brown-coloured). Scale bar, 10 mm. (**h**) IL-18 mRNA expression in liver biopsies from HCV patients (*n*=55) relative to uninfected control liver samples is shown (*n*=6). Bars represent means±s.e.m. Statistical significance was determined with the Mann–Whitney test (**b**,**d**,**h**) or the Wilcoxon matched-paired test (**a**,**c**). ns>0.05, *P 0.05, ***P≤0.001, ****P≤0.0001. conv, convalescent; HC, healthy control.

Having identified IL-18 as an important cytokine for viral MAIT cell activation *in vitro*, we further explored its relevance *in vivo*. In DENV infection, IL-18 levels in plasma were markedly increased during the acute phase compared to the convalescent phase of infection and healthy controls (Fig. 5a). In addition, patients suffering from the severe form of dengue (DHF) had significantly higher levels of IL-18 over the course of acute infection compared to patient with DF (Fig. 5b). Interestingly, expression of the IL-18 receptor (IL-18R) on MAIT cells was upregulated during the acute phase of infection and resolved to healthy control levels in the convalescent samples (Fig. 5c). As observed for IL-18, patients with DHF revealed higher expression of IL-18R on MAIT cells compared to patient with DF (Fig. 5d,e). Thus, levels of IL-18 in plasma and expression of IL-18R on MAIT cells *in vivo* are upregulated during the acute phase of infection, especially in those patients suffering from DHF. These findings show an association with increased activation of MAIT cells and disease severity in dengue patients (Figs 1f and 5f). These results are not restricted to DENV infection since IL-18 protein (Fig. 5g) and mRNA (Fig. 5h) were found readily in liver tissues from patients with HCV infection.

Overall, these data indicate that IL-18 is not only a dominant factor during *in vitro* viral MAIT cell activation, but also has an important role *in vivo*.

### MAIT cells respond to type I IFNs *in vitro* and *in vivo*

Type I IFNs are also important cytokines known for their strong antiviral activity. Previous reports have determined a crucial role of IFN-α/β during DENV^32^, influenza^33^ and HCV^34^ infections in limiting viral replication and disease severity. We therefore addressed whether IFN-α/β can also influence MAIT cell activation *in vitro* and *in vivo*.

**Figure 6:**
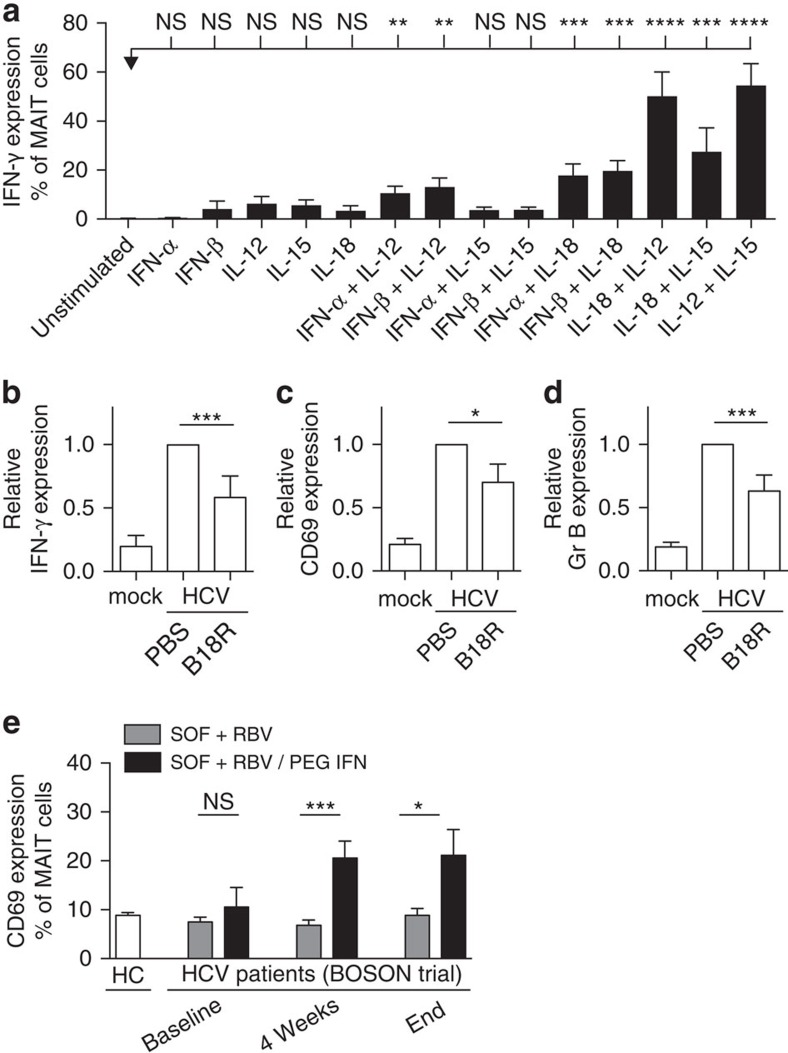
MAIT cells respond to type I interferons *in vitro* and *in vivo*. (**a**) PBMC's from healthy individuals were directly stimulated for 24 h with IFN-α, IFN-β, IL-12, IL-15, IL-18 or indicated combinations thereof. IFN-γ expression by MAIT cells (gated on live CD3^+^CD8^+^CD161^++^Vα7.2^+^ cells) was analysed by flow cytometry. (**b**–**d**) B18R (IFN-α/β neutralizing protein) or PBS control were added to the co-culture (*n*=6–11). IFN-γ (**b**), CD69 (**c**) and Granzyme B (**d**) expression is shown relative to the control. Data are representative from at least two independent experiments. (**e**) PBMC's from healthy controls or HCV patients at baseline, during or end of treatment with either SOF+RBV or SOF+RBV/PEG-IFN were analysed by flow cytometry. CD69 expression on MAIT cells (gated on live CD3^+^CD8^+^CD161^++^Vα7.2^+^ cells) was measured. Bars represent means±s.e.m. Statistical significance was determined with the Kruskal–Wallis test followed by the Dunns' test (**a**) or the Mann–Whitney test (**b**–**e**). ns>0.05, *P 0.05, ***P≤0.001, ****P≤0.0001. HC, healthy controls.

First, we tested whether IFN-α/β could trigger MAIT cells alone or in combination. We found IFN-γ secretion by MAIT cells in response to IFN-α/β in combination with IL-12 and IL-18, but not IL-15 (Fig. 6a), in a dose-dependent manner (Supplementary Fig. 7). Next, we confirmed that these TCR-independent responses to cytokines are shared with other CD161^++^ T-cell subsets, but not CD161^−^ T-cell populations. CD161^++^CD8^+^ T cells that lack expression of the Vα7.2Jα33 TCR (Supplementary Fig. 8a), CD161^++^ Vα7.2Jα33^+^ CD4 T cells (Supplementary Fig. 8b) and CD4^−^CD8^−^ T cells (Supplementary Fig. 8c) could all be triggered in response to cytokines, unlike CD161^−^ T-cell populations (Supplementary Fig. 8d). This is in agreement with our previous findings which suggest that CD161-expressing lymphocytes display a shared innate response to cytokines^16^.

To further explore the role of type I IFNs during viral MAIT cell triggering, we used a vaccinia virus-encoded, soluble type I IFN receptor (B18R)^35^. Macrophages treated with HCV *in vitro* were incubated in the presence of B18R and T cells overnight. Blockade of type I IFNs by B18R inhibited MAIT cell IFN-γ secretion (Fig. 6b), CD69 expression (Fig. 6c) and Granzyme B upregulation (Fig. 6d).

IFN-α has been an important component of HCV treatment. This gave us an opportunity to explore whether type I IFNs could contribute to MAIT cell activation *in vivo*. We measured the activation marker CD69 on MAIT cells from HCV patients taking part in a clinical trial in which they were treated with either sofosbuvir (SOF)+ribavirin (RBV)+pegylated IFN (PEG-IFN), or only SOF+RBV, without PEG-IFN. Interestingly, CD69 expression on MAIT cells was upregulated only in the treatment group receiving PEG-IFN (Fig. 6e), which corresponded with a significantly higher sustained virologic response rate^36^. High CD69 upregulation was specific to CD161^++^MAIT cells and not observed on CD161^−^CD8^+^ or CD4^+^ T cells (Supplementary Fig. 9). These data demonstrate that type I IFNs can contribute to MAIT cell activation *in vitro* and *in vivo.*

### Activated MAIT cells can limit HCV replication

**Figure 7:**
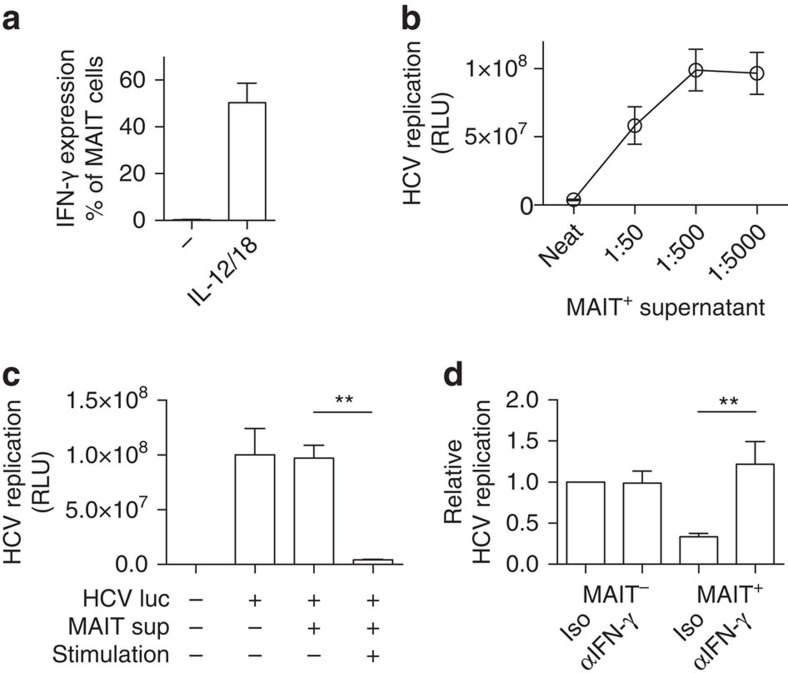
Activated MAIT cells can limit HCV replication. Sorted MAIT cells (CD8^+^CD161^++^Vα7.2^+^) were rested or activated in a TCR-independent manner (IL-12+IL-18 stimulation, MAIT^+^) for 24 h. (**a**) IFN-γ expression by MAIT cells (gated on live CD3^+^CD8^+^CD161^++^Vα7.2^+^ cells) was analysed by flow cytometry. (**b**,**c**) Neat or diluted supernatants were transferred to hepatocyte lines infected with HCV expressing luciferase and viral replication measured 4 days post infection. (**d**) Supernatants were transferred to hepatocyte lines infected with HCV expressing luciferase repeated in the presence of an isotype control or anti-IFN-γ antibody. Data are representative from at least two independent experiments. Bars represent means±s.e.m. Statistical significance was determined with a Wilcoxon matched-paired test (**c**) or the Mann–Whitney test (**d**). **P≤0.01. −, unstimulated; luc, luciferase; RLU, relative light units; sup, supernatant.

Finally, we explored whether viral MAIT cell activation can have a functional impact on virus replication. We stimulated sorted MAIT cells in a TCR-independent manner (IL-12 and IL-18) and confirmed activation by measurement of IFN-γ secretion (Fig. 7a). The cell supernatants of unstimulated and stimulated MAIT cells were transferred to a hepatocyte line infected with HCV expressing luciferase, allowing measurement of HCV replication. The activated MAIT cell supernatants were able to potently suppress HCV replication, while no activity from control supernatants (Fig. 7b) or from IL-12 and IL-18 (data not shown) addition to hepatocytes was seen. Diluting the activated MAIT cell supernatant reduced the suppression of HCV replication in a dose-dependent manner (Fig. 7c). Addition of anti-IFN-γ reversed the HCV suppression by activated MAIT cell supernatants (Fig. 7d). In an extension of the experimental set up using virally-induced activation to trigger MAIT cells, supernatants from MAIT cells incubated with HCV-treated macrophages reduced HCV replication in the hepatocyte reporter cell line compared to mock-treated macrophages (Supplementary Fig. 10). These results indicate that activation of MAIT cells by viral triggers induces responses that can impact on viral replication *in vitro.*

## Discussion

MAIT cells are an abundant human T-cell population with a semi-invariant TCR determining bacterial specificity. It has previously been considered that MAIT cells were not responsive to viral infection^37^. However, in this study we provide data to show MAIT cells are also readily and specifically activated by pathogenic viruses *in vivo* and *in vitro*. Furthermore, we define mechanisms that allow this virally-driven activation, and show they are distinct in the different viral settings.

The *in vivo* data shows that there is evidence of MAIT cell activation in response to DENV, HCV and influenza virus. Of these the most striking is the data from acute DENV infection as it is clear that such activation is profound and rapid in this severe clinical setting. Of note, those patients who went on to develop DHF exhibited significantly higher levels of activation as judged by CD38 expression. This suggests the MAIT cell response could contribute to the pathophysiology of this condition. The correlation between levels of IL-18, levels of IL-18R and MAIT cell activation suggests an important role for IL-18 in this activation process, consistent with the *in vitro* mechanisms demonstrated. In DENV, HCV and influenza virus infection we found clear evidence of activation of the MAIT cells, as judged by upregulation of Granzyme B (which is tightly regulated in MAIT cells^22^). In addition, we noted a decrease in circulating MAIT cell frequencies. The mechanism for this is not clear. It may result from translocation of some cells from blood to tissues, or it could result from activation-induced cell death as has been suggested for HIV infection^38^.

The data presented are correlative—however probing a non-redundant role in animal models is not straightforward. In mice the MAIT cell population is a very small minority compared to the frequencies of iNKT cells, which have overlapping functions and innate cytokine responsiveness^39^. In humans, the situation is reversed, with MAIT cell frequencies exceeding those of iNKT cells by 1–2 logs^13^. Existing data indicate a functional antiviral role for iNKT cells in control of virus infections in tissues such as lung, in mouse models: given similar molecular mechanisms of activation apply, and similar effector functions are induced, it seems reasonable to extrapolate from this that MAIT cell activity would have a parallel role in human infection^40^. Emerging models with increased MAIT cell frequencies in mice may help address this question using new *in vivo* approaches^41^.

MR1 tetramers unequivocally identify MAIT cells, but these reagents are not yet widely available. In this study we used CD161^++^Vα7.2^+^ as a surrogate marker for MAIT cells, which is a commonly used staining approach and includes the majority of tetramer-positive MAIT cells^6^,^42^. We further defined MAIT cells as CD8^+^CD4^−^CD3^+^ T cells, and for *in vitro* experiments PBMC's from healthy individuals were used. CD8 is expressed by the majority of MAIT cells, and CD4 only by a small proportion of MAIT cells^42^. This approach has been used in recent transcriptional studies and allows ready comparison with related CD161-expressing subsets^16^. Future studies using MR1 tetramers are needed to further establish the distribution of MAIT cell phenotype in virally infected humans in more detail. As we did not have access to the tetramer, the observed loss of MAIT cells in virally infected patients may be potentially explained by down-regulation of CD161, as previously suggested^12^,^43^. However, *Fernandez et al.*^44^ showed that in HIV-1 infected patients, MR1 tetramers do not bind CD161^−^Vα7.2^+^ T cells, and the loss of MAIT cells in the periphery may be due to recruitment to the site of infection or activation-induced cell death. Of note, our data are not restricted to CD161^++^Vα7.2^+^ cells but also includes responses from the minority CD161^++^Vα7.2^−^ cells, which we have previously described as sharing phenotypic and functional properties with MAIT cells and which likely also include some recently described Vα7.2^−^ MR1-restricted T cell populations^45^,^46^.

The mechanisms we have explored reveal a central role for IL-18 in virus-induced activation^12^,^16^. However, the critical co-stimulation was not restricted to a single cytokine. In the case of HCV we repeatedly observed an important role for IL-15. IL-15 levels in these experimental conditions were very low, but upregulation of IL-15R was seen and importantly antibody-blockade of IL-15 revealed a clear role for this cytokine. In addition, we observed a role for type I IFNs in the activation of MAIT cells in response to HCV. This was clear in both addition and blockade experiments and is consistent with data from natural killer cells, where such cytokine-mediated activation is described^47^.

The cytokine activation of MAIT cells was mediated by APCs with a role for viral sensing and replication. In the case of DENV, viral replication was required for MAIT cell activation and this was positively correlated with the level of productive infection in the APC. Although we observed a reduction of MAIT cell activation in response to UV-irradiated HCV and IAV, replication does not have a key role in this context, since HCV and influenza poorly replicate in macrophages^28^,^29^. As APC have continual and extensive endocytic activity, viral recognition can occur through endocytosis of viral particles as well as through direct infection^26^,^48^. Using TLR agonists, we found that both surface and endocytic TLRs can trigger MAIT cells, in particular viral pathogen-associated molecular patterns.

Using murine bone marrow-derived DCs, Le Bourhis *et al*.^37^ has previously shown that MAIT cells are not responsive to viral infection. This discrepancy is likely due to the use of murine, rather than human cells, which may reflect differential abilities in secreting, as well as, responding to cytokines.

IFN-α-mediated activation of MAIT cells is clinically and functionally relevant, as demonstrated *in vivo* in the well-controlled BOSON clinical trial. In addition to directly acting antiviral drugs (SOF in combination with RBV), IFN-α was included in one arm only and associated with MAIT cell activation and an enhanced therapy response^36^. It is possible that MAIT cells act as local amplifiers of IFN-α-mediated therapies, contributing to an antiviral effect through secretion of further local antiviral cytokines or potentially via clearance of infected cells. The specific activation by type I IFNs appears to be important evidence in favour of an evolution of MAIT cells in response to viral signals. Although type I IFN signalling is not exclusive to virus infections, a wealth of data links IFN pathways with antiviral defence, and we propose that MAIT cells form an important part of this network in humans. Of note, IFN-α signalling alone was insufficient to fully activate MAIT cells, indicating a potentially fail-safe mechanism to prevent harmful over-activation.

The secondary consequences of MAIT cell activation could be of significance for both protection and immunopathology. Activation of MAIT cells in tissues could provide both direct and indirect antiviral effects. HCV, for example, is known to be highly sensitive to T-cell-derived IFN-γ^49^,^50^. Accordingly, we found that activation of MAIT cells by HCV-induced responses that limited HCV replication *in vitro*, in an IFN-γ dependent manner. Thus, the local activation of MAIT cells can be plausibly correlated with a direct antiviral function *in vivo*.

Loss of MAIT cell frequencies (as we have observed in blood) may contribute to impairment of both viral and bacterial control, whereas excess cytokine secretion by MAIT cells may lead to immunopathologic outcomes. For example, high levels of MAIT cell activation could contribute to the cytokine ‘storm' associated with DHF and severe cases of influenza virus^51^,^52^. Activation-induced loss of MAIT cells is potentially relevant in the context of influenza virus infection, which is epidemiologically associated with secondary bacterial pneumonia due to *Staphylococcus aureus* or *Streptococcus pneumonia*^53^,^54^. Both of these organisms possess the MAIT cell ligand^7^,^37^, and it is plausible that depletion of MAIT cells *in situ* could lead to transient impairment of local control of these pathogens. Interestingly, the transcription factor PLZF, which likely drives the innate phenotype and functionality of MAIT cells, may also contribute to the induction of activation-induced cell death, through upregulation of specific intracellular caspases^55^.

In conclusion, human MAIT cells are activated in response to virus infections (as illustrated by a simplified model depicted in Supplementary Fig. 11), through overlapping mechanisms dependent on the virus and likely the environment and cell types affected. These data extend the likely functions of these abundant human lymphocytes well beyond their traditional confines. Modulation of this cell subset (and related CD161^+^ T-cell subsets^16^,^56^,^57^,^58^) could provide a novel opportunity to promote antiviral defence or limit immunopathology.

## Methods

### Patient samples

All samples were collected with appropriate patient consent and local research ethics committee approval. Samples were frozen and stored at −80 °C until examined. Healthy donors were supplied by the NHS blood service without any further donor information. The study on DENV patient samples was approved by the Scientific and Ethical Committee of the Khon Kaen Hospital in Thailand and the Riverside Ethics Committee in the UK (06/Q0401/22). Laboratory confirmation of DENV infection was determined by RT–PCR detection of DENV nucleic acid (which also confirmed the infecting serotype) or seroconversion in an ELISA of IgM. Secondary infection was defined based on the ratio of DENV-specific IgM to IgG <1.2 on or after day 6 of illness. Disease severity was classified according to 1997 World Health Organization criteria. Of the patients enroled in the study, 10 patients were classified as mildly symptomatic of dengue fever and 10 patients were classified as severely symptomatic of dengue haemorrhagic fever with plasma leakage and bleeding (Supplementary Table 1). The day of defervescence was defined as day 0, the day before defervescence as day −1, the day after defervescence as day +1, and so forth. PBMCs were isolated from whole blood by Ficoll-Hypaque density-gradient centrifugation at various time points during hospitalization and cryopreserved until further use.

Patients with influenza virus infection requiring hospitalization were recruited from two hospitals based in Oxford and Glasgow during the 2009 H1N1 pandemic under ethical approval (09/H0606/92). Influenza virus infection was diagnosed using an in-house generic influenza A real time PCR assay based on the Matrix gene and confirmed with swine-origin H1N1/09 specific Matrix gene PCR^59^,^60^. Patients had concomitant conditions as documented in Supplementary Table 2, but were not on immunosuppressants or steroids at the point of sampling. Two patients were pregnant, and one was 6 days post-partum. PBMC samples from HCV patients (Supplementary Table 3) were collected from the Hepatitis Clinic at the John Radcliffe Hospital, Oxford, UK and consented according to a locally approved protocol (COREC 04.OXA.010). Liver biopsy specimens were obtained from patients with HCV infection (*n*=55) at S. Bortolo Hospital, Vicenza, Italy and scored using the Ishak system. Control samples were normal adjacent tissue from six uninfected volunteers undergoing liver resection for other reasons (source Proteogenex, CA, USA). Trials were conducted in accordance with the Declaration of Helsinki, ethical approval was obtained from local ethics committees, and all patients provided written informed consent. Explanted HCV-diseased liver tissues were obtained from QE transplant programme and non-diseased normal liver tissue was obtained from donor liver tissue surplus to clinical requirements. Normal liver was liver tissue from hepatic resection of colorectal metastasis. For studying MAIT cell activation in the context of HCV infection and type I IFNs *in vivo*, PBMC from the BOSON clinical trial were used, as part of a collaboration between STOP-HCV and Gilead Sciences^36^,^61^. BOSON is a randomized, open-label, phase 3 study testing SOF plus RBV with or without pegylated IFN-α in patients with HCV genotype 3 and treatment-experienced cirrhotic patients with HCV genotype 2 (Supplementary Table 4). This study was registered with the European Clinical Trials Database, number 2013-002641-11.

### Tissue staining

Paraffin-embedded human liver tissues sections were dewaxed using Clearene and IMS. Endogenous peroxidase was blocked by using 5% hydrogen peroxide followed by antigen retrieval using 10% EDTA buffer. Slides were blocked using casein buffer. Primary Rabbit IL-18 antibody LS-B2809, 1:50 dilution was applied for an hour followed by Vector Impress anti-rabbit secondary for 30 min in a staining chamber with rocking. Slides were then washed with PBS+Tween and Impress DAB substrate was applied for 2½ mins. Slides were then counterstained in Mayer's haematoxylin and mounted in DPX.

### Cell culture

Human PBMCs were isolated from leukocyte cones from healthy donors supplied by the NHS blood service. CD8+ T-cells were isolated from PBMCs using positive selection with MACS CD8+ microbeads (Miltenyi). Monocytes were enriched from PBMCs using MACS CD14+ microbeads (Miltenyi). Monocytes were differentiated into macrophages (macrophages or GM-MΦ), by incubating the CD14+ cells for 7 days in X-VIVO15 (Lonza), Pen/strep (Sigma-Aldrich), L-glutamine (Sigma-Aldrich) with 50 ng ml^−1^ GM-CSF (Miltenyi). Immature dendritic cells (imDC's or DC's) were obtained by culturing CD14^+^ cells for 4–5 days in the presence of 20–100 ng ml^−1^ GM-CSF (Miltenyi/First Link) and 25 ng ml^−1^ IL-4 (Miltenyi/eBioscience). Where stated, MAIT cells were sorted using a Beckman Coulter MoFlo XDP.

### Viruses

DENV serotype 2, strain 16681, was grown in C6/36 cells in Leibovitz's L-15 medium with L-glutamine and supplemented with 2% fetal calf serum. Culture medium was centrifuged and stored at −80 °C. The titres of virus were determined by a focus-forming assay on Vero cells and expressed as focus-forming units per ml. Briefly, virus was serially diluted and incubated with Vero cells for 2 h at 37 °C. The monolayers were then overlaid with 1.5% carboxymethylcellulose and incubated at 37 °C for 3 days. Virus foci were stained with anti-DENV E antibody (4G2) followed by peroxidase-conjugated anti-mouse Ig and visualized by the addition of DAB substrate. Infection rates of DENV infected DCs were assessed using an antibody detecting the non-structural DENV protein, NS3, by flow cytometry. The mouse monoclonal anti-DENV NS3 (E1D8) and anti-DENV E (4G2) were gifts from E. Harris and AFRIMS.

A total of 10 ng RNA transcribed from genotype 2a HCV strain J6CF-JFH1 (obtained from Prof. Bartenschlager^62^) was electroporated into Huh-7.5 cells (obtained from Apath) and were cultured for up to 3 weeks. Cell culture supernatants were collected for up to 20 days post electroporation centrifuged and concentrated using an Amicon Ultra-15 (Millipore). This inoculum was used to infect huh-7.5 cells. The HCV titre was determined by immunofluorescence. Huh-7.5 cells were fixed with methanolacetone, blocked with BSA/phosphate-buffered saline (PBS) solution, washed with PBS, stained with anti-HCV core primary antibody (Cambridge Biosciences), washed with PBS and Alexa Fluor 488 donkey polyclonal secondary antibody to Mouse IgG (ab150105, Abcam). To measure HCV replication a HCV luciferase reporter Jc1FLAGp7-nsGluc2A^29^ virus was used. Jc1FLAG(p7-nsGluc2A) was grown in naïve Huh-7.5 cells^63^ and the infectivity titre was determined by limiting dilution titration on naive Huh-7.5 cells as median tissue culture infective dose (TCID50). To measure HCV replication, Huh-7.5 cells were plated in a 96-well plate and infected at an MOI of 0.1 for 6 h. After washing the Huh-7.5 cells, supernatants from (un)stimulated MAIT cells were added in the presence or absence of a neutralizing IFN-γ (clone MD-1, 14-7317-85 eBioscience). Supernatants were collected 4 days post infection and luciferase expression determined using the Renilla Luciferase Assay System (Promega) and a Berthold TriStar2 multimode reader LB 942.

Influenza A/WSN/33 (H1N1) virus (WSN) was grown in Madin Darby Bovine Kidney (MDBK, obtained from the European Collection of Cell Cultures) cells in Minimum Essential Medium Eagle (Sigma) with 2 mM L-glutamine and 0.5% fetal calf serum. To produce virus stocks, six 175 cm^2^ tissue culture flasks containing sub-confluent MDBK cells were infected at low-multiplicity. After 48 h of culture in a 37 °C humidified incubator, supernatants were collected and clarified by low-speed centrifugation (30 min, 2,000 *g* and 30 min, 18,000 *g*, 4 °C). Next, WSN was concentrated through a 30% sucrose cushion by ultracentrifugation (90 min, 112,000 *g*, 4 °C). Finally, WSN was purified on a 30–60% sucrose gradient by ultracentrifugation (150 min, 209,000 *g*, 4 °C). The visible band of virus was drawn off with a needle. (Hutchinson, E. and Fodor, E., Nature ProtocolExchange, Purification of influenza virions by haemadsorption and ultracentrifugation, ‘ http://www.nature.com/protocolexchange/protocols/3315')^64^,^65^. Plaque assays were performed on MDBK cells using standard techniques. Influenza A (H3N2) virus A/England/691/2010 is a clinical strain isolated by Public Health England (PHE), as part of the MOSAIC project. Influenza B virus, B/Florida/04/2006 was derived from reverse genetics systems by *de novo* synthesis (GeneArt).

### *In vitro* virus experiments

Unless specified differently, for co-culture experiments using viruses, APCs were treated with virus at a MOI of 1, as determined based on the viral titre and number of cells plated, for 90 to 120 min at 37 °C. Virus was washed off and isolated CD8+ T-cells were added for an additional 10–24 h, unless specified differently. In the case of the DENV experiments, PBMC's were added instead of isolated CD8+ T-cells. In the case of influenza virus, WSN virus was used, unless specified differently.

### *In vitro* cytokine stimulations

For cytokine stimulation experiments, isolated CD8+ T-cells or PBMC's were stimulated for 24 h with 50 ng ml^−1^ IL-12 (Miltenyi Biotech), 50 ng ml^−1^ IL-18 (R&D Systems Europe), 50 ng ml^−1^ IL-15 (Miltenyi Biotech) or 2,000 U ml^−1^ IFN-α (Sigma-Aldrich) or 50 ng ml^−1^ IFN-β (Miltenyi Biotech) or combinations thereof. For blocking experiments, neutralizing agents were added into the culture together with the CD8^+^ T-cells or PBMC's. Blocking antibodies against IL-12p40/70 (508804, eBioscience), IL-12p70 (MAB219-100, R&D), IL-15 (MAB2471, R&D) or IL-18 (D044-3, MBL International, USA), IFN-γ (14-7317-85, eBioscience) or MR1 (361102, Biolegend) were used at 5–10 μg ml^−1^. Type I IFN was blocked using 1 μg ml^−1^ B18R (34-8185-81, eBioscience).

### Flow cytometry

Antibodies/dyes and dilutions used were: viability dye live/dead fixable-violet (L34955, Invitrogen, 1:1250), CD3-eFluor450 (48-0038, eBioscience, 1:100), CD3-PECy7 (25-0038, eBioscience, 1:100), CD4-VioGreen (130-096-900, Miltenyi Biotech, 1:50), CD8-VioGreen (130-098-062, Miltenyi Biotech, 1:50), CD8-V450 (560347, BD, 1:50), CD8-PerCP.Cy5.5/PerCP (301032, Biolegend, 1:100), CD38-APC (555462, BD, 1:50), CD69-FITC (11-0699, eBioscience, 1:40), CD161-APC (130-098-908, Miltenyi Biotech, 1:100), CD161-PE (130-099-193, Miltenyi Biotech, 1:100), IFN-γ-FITC (130-091-641, Miltenyi Biotech, 1:50), IFN-γ-APCCy7 (502529 Biolegend, 1:50), Vα7.2-PE/PeCy7/APC/FITC (351705/351711/351707/351703, Biolegend, 1:50). Granzyme B-APC (MHGB05, Invitrogen), IL-18Ra-APC (17-7183-41, eBioscience, 1:50), TNF-α-PeCy7 (502929, Biolegend, 1:100). All data was acquired on a MACSQuant (Miltenyi Biotech) or a BD FACSVerse (BD) and analyzed on FlowJo (Tree Star Inc.). Gating strategy is shown in Supplementary Fig. 12.

### Cytokine measurements

Cytokines levels in this study were measured from cell culture supernatants or heparin plasma samples. Cell culture supernatants from macrophages treated with mock, HCV or influenza A were collected at 48 h. Cell culture supernatants from DCs treated with mock or DENV were collected at 42 h. Heparin plasma samples were collected from DENV infected patients at different phases of illness or from healthy controls. Cell culture supernatants and heparin plasma samples were frozen at −80̊C until further use. Cytokine levels were analysed using Bio-Plex human cytokine kits (BioRad) and acquired on a Luminex 100 (Luminex) or a Bio-Plex 200 reader (BioRad) according to the manufacturer's instructions.

### Statistical analysis

Statistical analysis was carried out with GraphPad Prism software. Statistical significance was reported as ns *P*>0.05; *P≤0.05; **P≤0.01; ***P≤0.001; ****P≤0.0001. Error bars on graphs represent s.e.m. Mann–Whitney test was used to calculate significance levels between two groups. Wilcoxon matched-paired test was used to calculate significance levels between two paired groups. For comparisons of means from multiple groups against one control group the Kruskal–Wallis with Dunn's multiple comparison post-test analysis was performed. Spearman-rank correlation analysis was used to calculate correlations. Sample sizes were adequate to detect large effects between groups, as determined by the reproducibility and variability of each particular experiment and limited by the availability of patient samples. No randomization or blinding was used.

## Additional information

**How to cite this article:** van Wilgenburg, B. *et al*. MAIT cells are activated during human viral infections. *Nat. Commun.* 7:11653 doi: 10.1038/ncomms11653 (2016).

## Supplementary Material

Supplementary Information Supplementary Figures 1-12 and Supplementary Tables 1-4
