# Supplementary material for: MAIT cells are activated during human viral infections
Source: Nat Commun. 2016 Jun 23;7:11653. doi: 10.1038/ncomms11653 (PMC4931007; doi:10.1038/ncomms11653)
Supplement: Supplementary Information — Supplementary Figures 1-12 and Supplementary Tables 1-4 [file ncomms11653-s1.pdf]

Supplementary Figures

Supplementary Figure 1

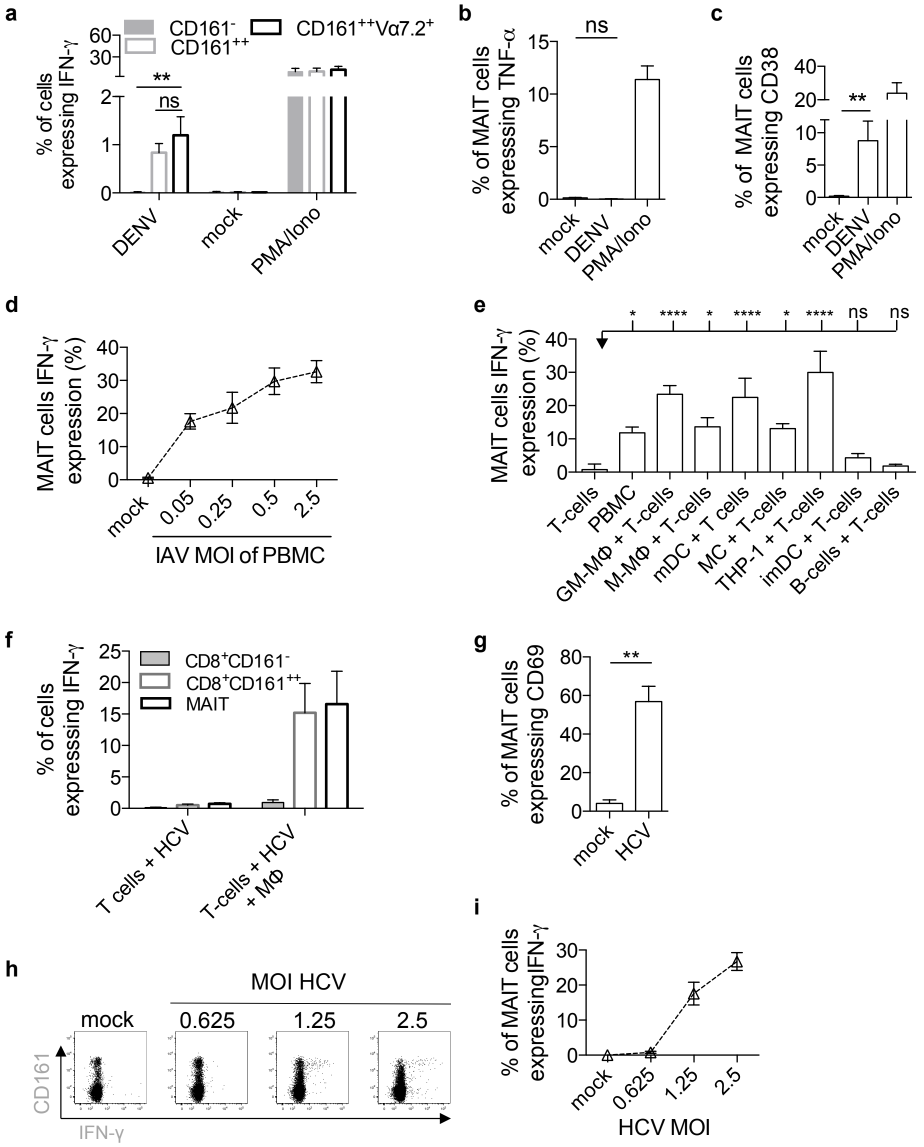

Supplementary Figure 1. Specific MAIT cell activation in response to viruses.

(**a**, **b,** **c**) PBMC's from healthy individuals were co-cultured with autologous monocyte derived dendritic cells (DC's) exposed to Dengue Virus (DENV). Proportion of indicated cell population (n=4-8) expressing IFN-γ (**a**) or TNF-α (**b**) or CD38 (**c**) in response to DENV or 100 ng/ml PMA and 500 ng/ml Ionomycin. (**d**) MOI-dependent activation of MAIT cells in an influenza virus-exposed PBMC culture (n=5-8). (**e**) Percentage of MAIT cells expressing IFN-γ in response to influenza virus in the following culture conditions: $CD8^+$ T-cells only (n=20), PBMC only (n=20), or $CD8^+$ T cells co-cultured with macrophages either cultured in the presence of GM-CSF (MΦ or GM-MΦ (n=15)) or M-CSF (M-MΦ (n=15)), mature Dendritic Cells (mDC (n=15)), monocytes (MC (n=15)), THP-1 cells (n=13), immature DC's (imDC (n=15)) or B-cells (n=10). (**f**) $CD8^+$ T cells isolated from PBMC's from healthy individuals were co-cultured with exposed to HCV in the presence (n=11) in the absence of macrophages. IFN-γ expression by indicated cell population. Data are representative from at least two independent experiments. Bars represent means ± s.e.m. (**g-i**) $CD8^+$ T cells isolated from PBMC's from healthy controls co-cultured with macrophages exposed to HCV as described in "methods", unless indicated otherwise. (**h**, **i**) Increasing HCV MOI. (**h**) Representative flow cytometry plot. Percentage of CD69 (**f**, n=4-12) and IFN-γ (**i,** n=4-12) expression by MAIT cells. All data are representative from at least two independent experiments. Symbols and bars represent means ± s.e.m. Statistical significance Kruskal-Wallis test followed by Dunns' test (**g,** **j**). ns > 0.05, P 0.05, ****P ≤ 0.0001.

Supplementary Figure 2

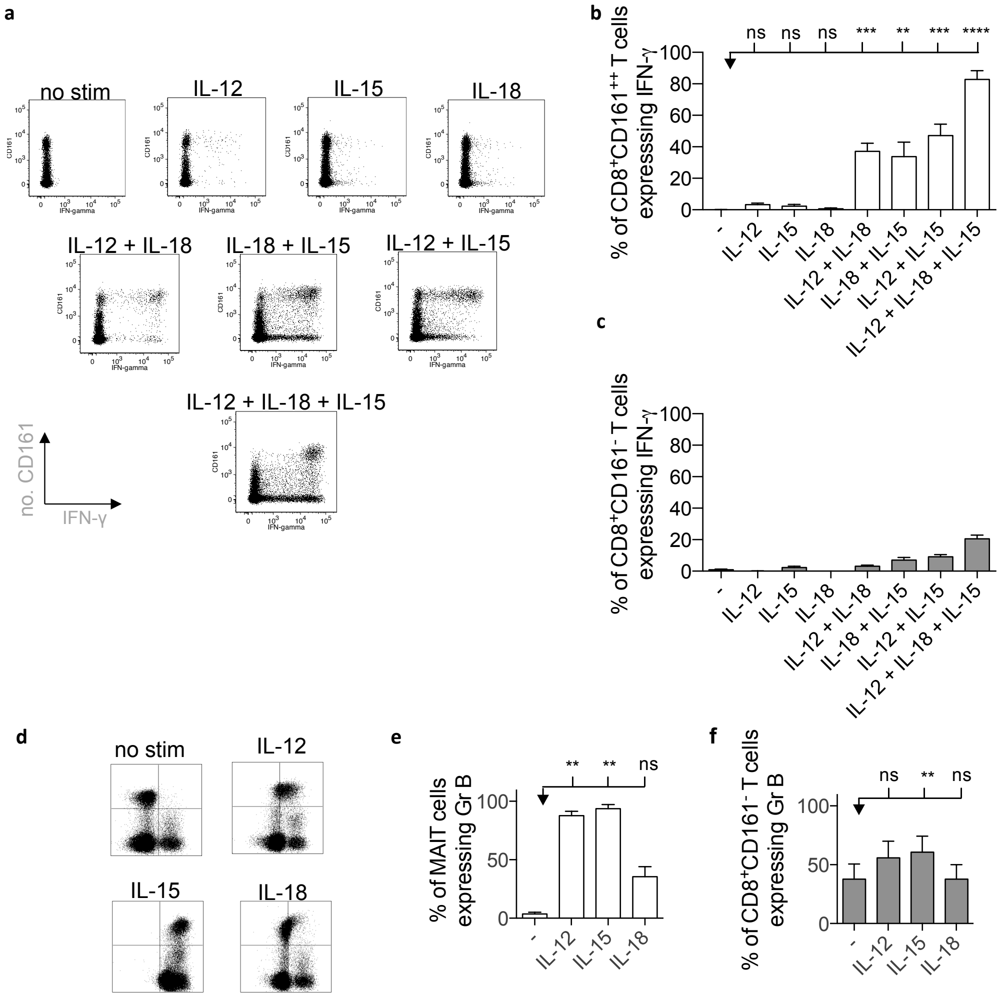

Supplementary Figure 2. Triggering of MAIT cells by cytokines.

(**a**-**c**) PBMC's (n=8) were stimulated for 24 h with the indicated cytokines, or combinations thereof. IFN-γ expression in $CD161^{++}CD8^{+}$ T cells (**b**) and $CD161^{-}CD8^{+}$ T cells (**c**) analysed by flow cytometry. (**a**) Representative plots. (**d**-**f**) PBMC's (n=5) were stimulated for 6 days with the indicated cytokine and Granzyme B was analysed in MAIT cells (**e**), $CD161^{-}CD8^{+}$ T cells and MAIT cells (**f**). Representative dot plots (**d**). Bars represent means ± s.e.m. Statistical significance was determined with Kruskal-Wallis test followed by Dunns' test. ns > 0.05, **P ≤ 0.01, ***P ≤ 0.001, ****P ≤ 0.0001.

Supplementary Figure 3

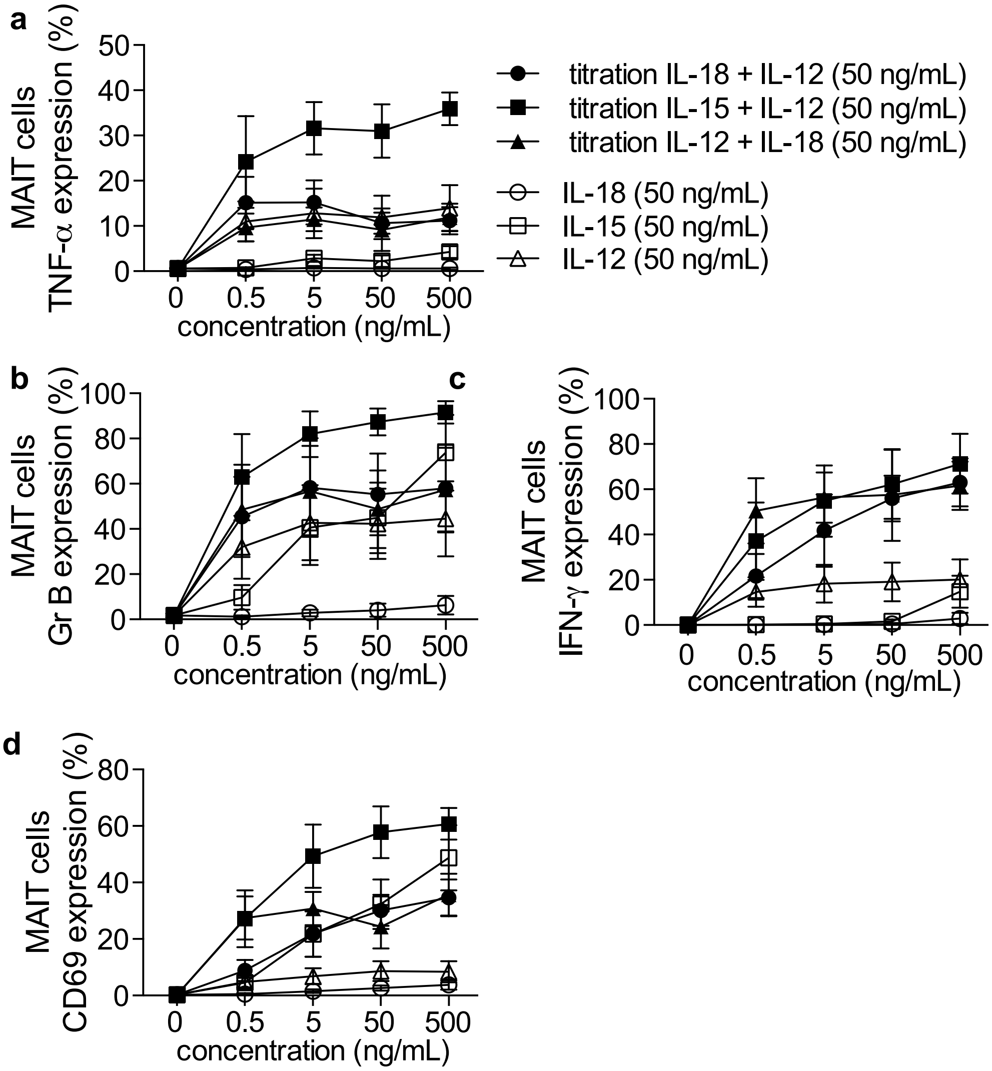

Supplementary Figure 3. Differences in MAIT cell responses in response to different cytokines. PBMC's from healthy individuals (n=3) were stimulated with the indicated concentration of a single cytokine (clear symbols) or combination of cytokines (black symbols – the concentration of one cytokine being fixed as indicated). Symbols represent mean ± s.e.m. (**a**) TNF-α, (**b**) Granzyme B, and (**c**) IFN-γ (**d**) CD69 expression by $CD3^{+}CD161^{++}V\alpha7.2^{+}$ cells.

Supplementary Figure 4

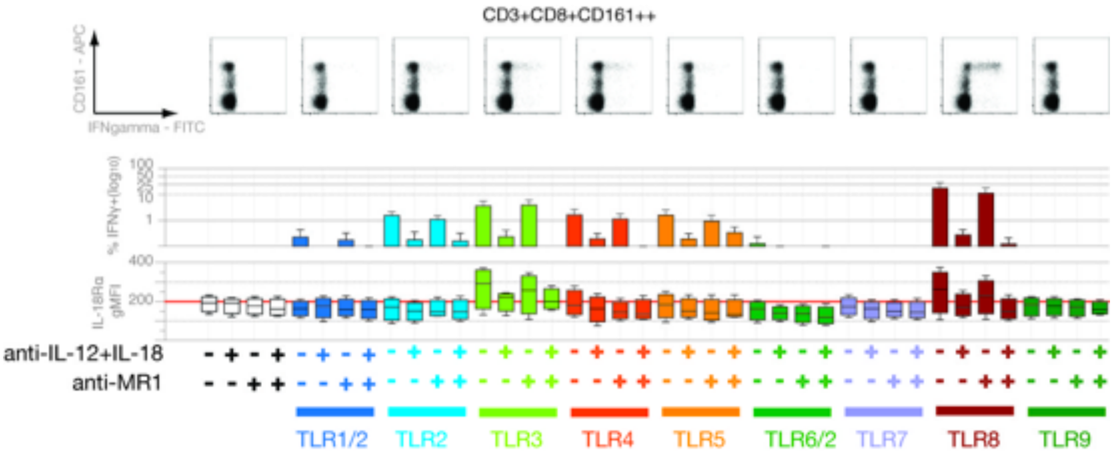

Supplementary Figure 4. Triggering of MAIT cells via toll-like receptors. PBMC's were cultured in the presence of TLR ligands for 24 h in the presence or absence of blocking antibodies again IL-12 and IL-18 or MR1. IFN-γ expression (top chart) and IL-18Rα (bottom chart) expression by MAIT cells were analysed by flow cytometry (gated on live $CD3^{+}CD8^{+}CD161^{++}V\alpha7.2^{+}$ cells). Representative dot plots of IFN-γ expression are shown in the top panel. Data are shown as mean ± SEM and are pooled from two experiments performed.

Supplementary Figure 5

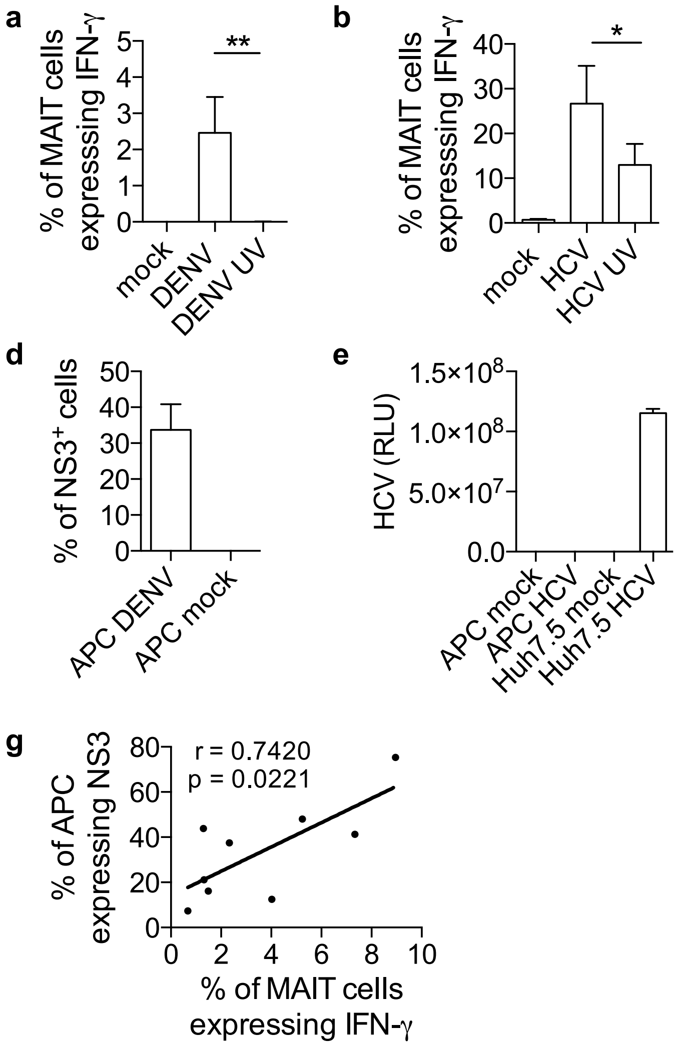

Supplementary Figure 5. UV-inactivation of viruses and MAIT cell activation.

UV inactivation of viruses was achieved using a UV lamp (Fisher Scientific UVS-18 EL hand held 220V 50Hz 8W 254 nm white light). Cells were exposed to (MOI=1): (**a**) DENV or UV-inactivated DENV (DENV UV) (n=5), (**b**) HCV or UV-inactivated HCV (HCV UV) (n=7), (**c**) Influenza A virus or UV-inactivated influenza A virus (n=10). (**d**) Infection rates of DENV infected APC's (n=9, MOI=1) were confirmed by intracellular staining of the non-structural DENV protein NS3 by flow cytometry. (**e**) APC's (n=4) and Huh7.5 cells (n=3) were treated with HCV expressing luciferase (MOI=0.1) and

luciferase expression was measured 4 days post infection (**f**). blood-derived APC's (n=3) or embryonic stem cell derived (es) APC's (n=3) or MDBK cells (n=1) were treated with Influenza A virus (MOI=2). Replication was determined using a plaque assay. (**g**) Correlation of infection rates of DENV infected APC's against MAIT cell IFN-γ expression by Spearman rank correlation test. Bars represent means ± s.e.m. Statistical significance was determined with Mann-Whitney test. ns > 0.05, *P 0.05, **P ≤ 0.01

Supplementary Figure 6

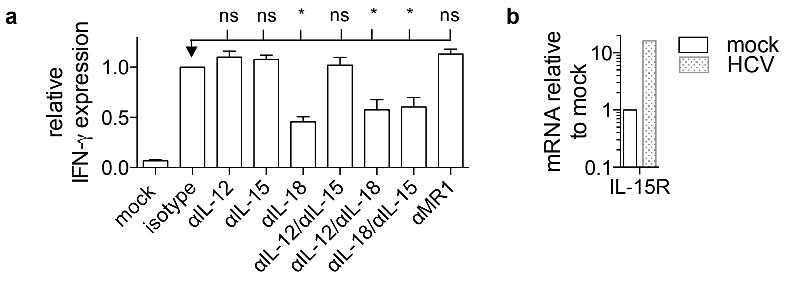

Supplementary Figure 6. Viral MAIT cells triggering is mediated by cytokines. (**a**) Influenza Virus-exposed PBMC culture as described in "methods". Isotype control, anti-MR1, anti-IL-12, anti-IL-15 and/or anti-IL-18 antibodies were added to the co-culture (n=5-29). IFN-γ expression is shown relative to the isotype control. Bars represent means ± s.e.m. Statistical significance was determined with Kruskal-Wallis test followed by Dunns' test. ns > 0.05, * P 0.05. (**b**) IL-15R mRNA expression in HCV-exposed macrophages relative to mock at 5 h.

Supplementary Figure 7

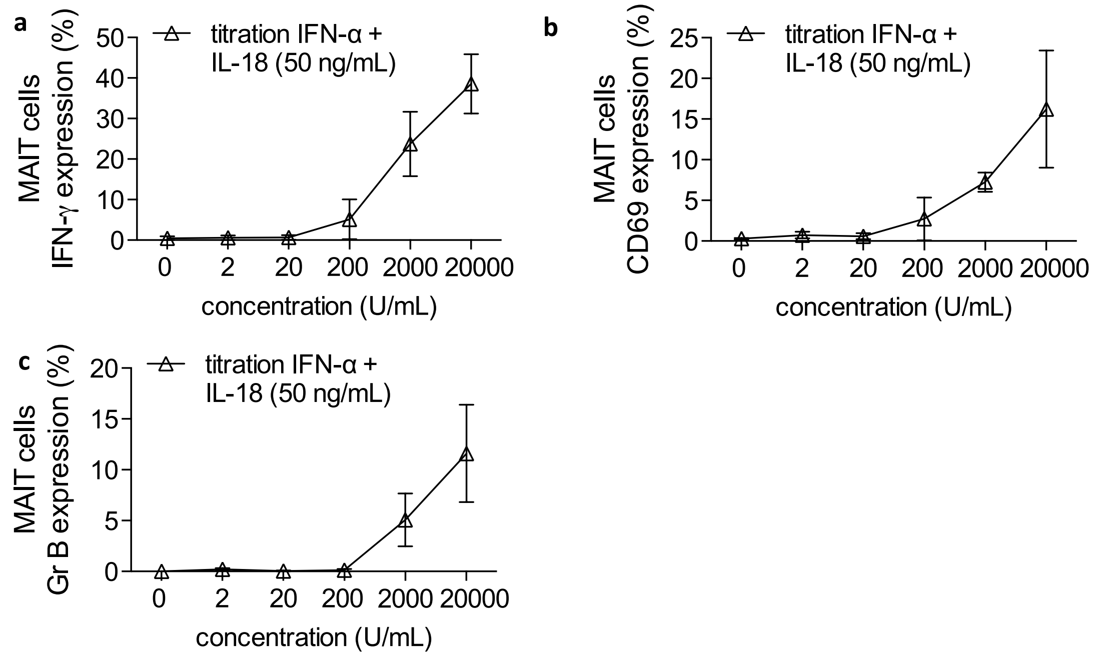

Supplementary Figure 7. Dose-dependent activation of MAIT cells by IFN-α. PBMC's from healthy individuals (n=3) were stimulated with the indicated concentration of IFN-α in combination with IL-18 (50ng/mL). Symbols represent mean ± s.e.m. (**a**) IFN-γ (**b**) CD69 expression (**c**) Granzyme B by $CD3^{+}CD161^{++}V\alpha7.2^{+}$ cells.

Supplementary Figure 8

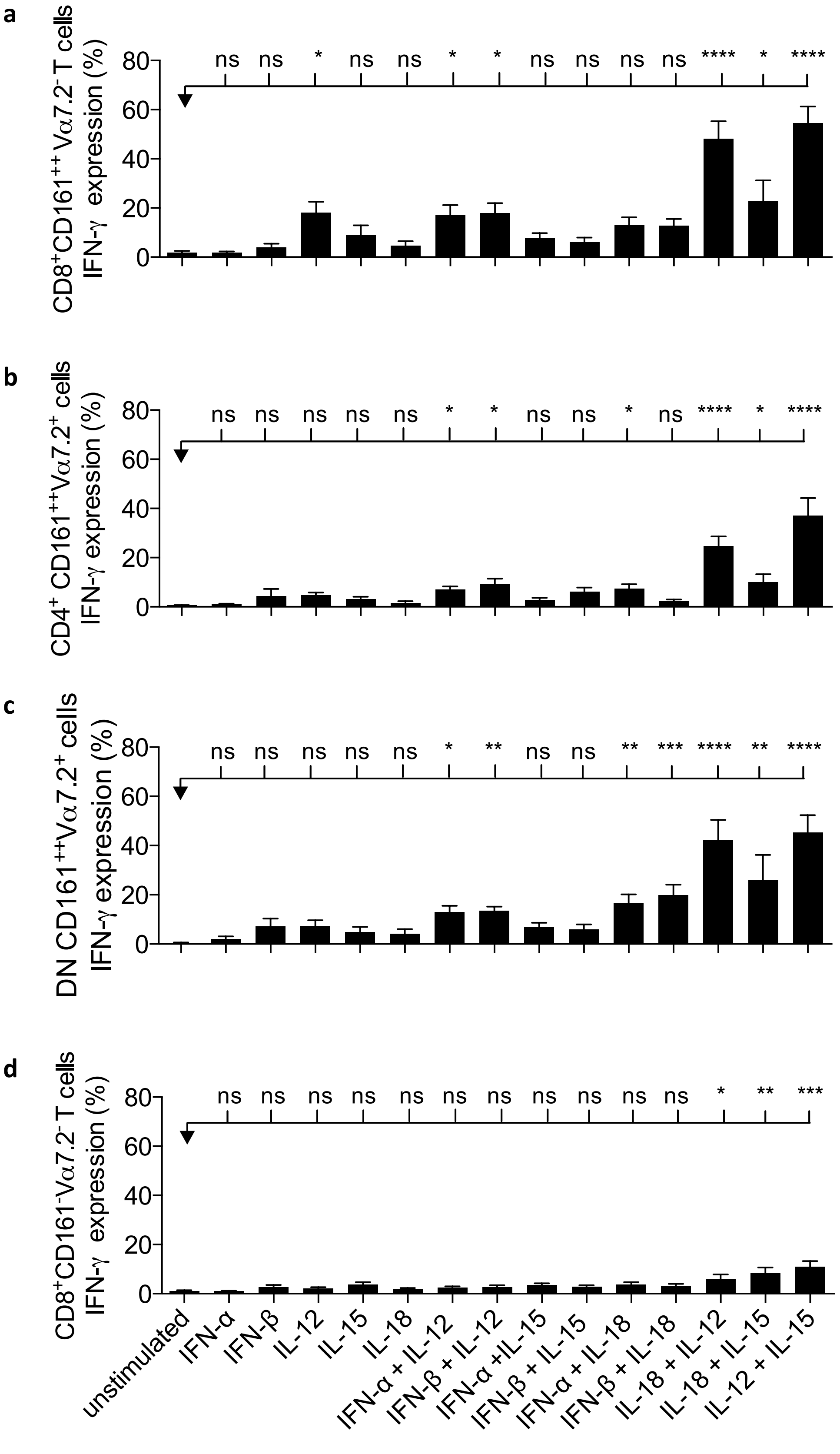

Supplementary Figure 8. Cytokine-mediated cell activation.

PBMC's (n=8) from healthy individuals were directly stimulated for 24 h (black bars) with IFN-α, IL-12, IL-15, IL-18 or indicated combinations thereof. Bars and symbols represent mean ± s.e.m. IFN-γ expression by $CD8^{+}CD161^{++}V\alpha7.2^{-}$ (**a**), $CD4^{+}CD161^{++}V\alpha7.2^{+}$ (**b**), $CD4^{-}CD8^{-}CD161^{++}V\alpha7.2^{-}$ (**c**), or $CD8^{+}CD161^{-}$ T cells (**d**). Statistical significance was determined with Kruskal-Wallis test followed by Dunns' test. ns > 0.05, *P 0.05, **P ≤ 0.01, ***P ≤ 0.001, ****P ≤ 0.0001.

Supplementary Figure 9

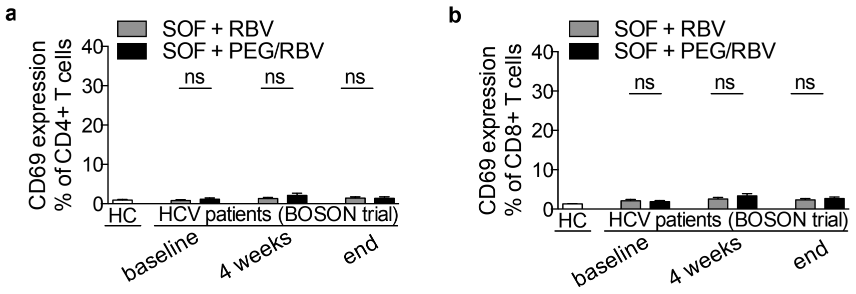

Supplementary Figure 9 Bulk CD4 and CD8 T cells do not respond to Type I Interferons *in vivo*. PBMC's from healthy controls or HCV patients at baseline, during or end of treatment with either SOF + RBV or SOF + RBV/PEG-IFN were analysed by flow cytometry. CD69 expression on $CD4^+$ T cells (**a**) or $CD8^+$ T cells (**b**) was measured. Bars represent means ± s.e.m. Statistical significance was determined with the Kruskal-Wallis test followed by Mann-Whitney test. ns > 0.05. Abbreviations: HC, healthy controls; PEG-IFN, pegylated interferon; RBV, Ribavirin; SOF, Sofosbuvir.

Supplementary Figure 10

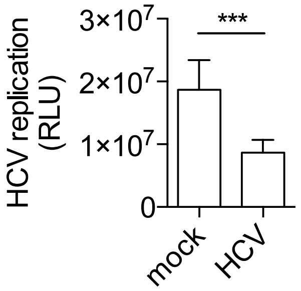

Supplementary Figure 10. Virally activated MAIT cells can limit virus replication. Mock or HCV-treated macrophages (MOI=1) were co-cultured with CD8^{+} T-cells from healthy individuals (n=4) for 24 h. Supernatants were transferred to hepatocyte lines infected with HCV expressing luciferase (MOI=0.1) and viral replication was measured after 96 hours. Data are from one experiment with three technical repeats. Bars represent means ± s.e.m. Statistical significance was determined with a Wilcoxon matched-paired test. ***$P \leq 0.001$. HCV, Hepatitis C Virus; RLU, Relative Light Units.

Supplementary Figure 11

a

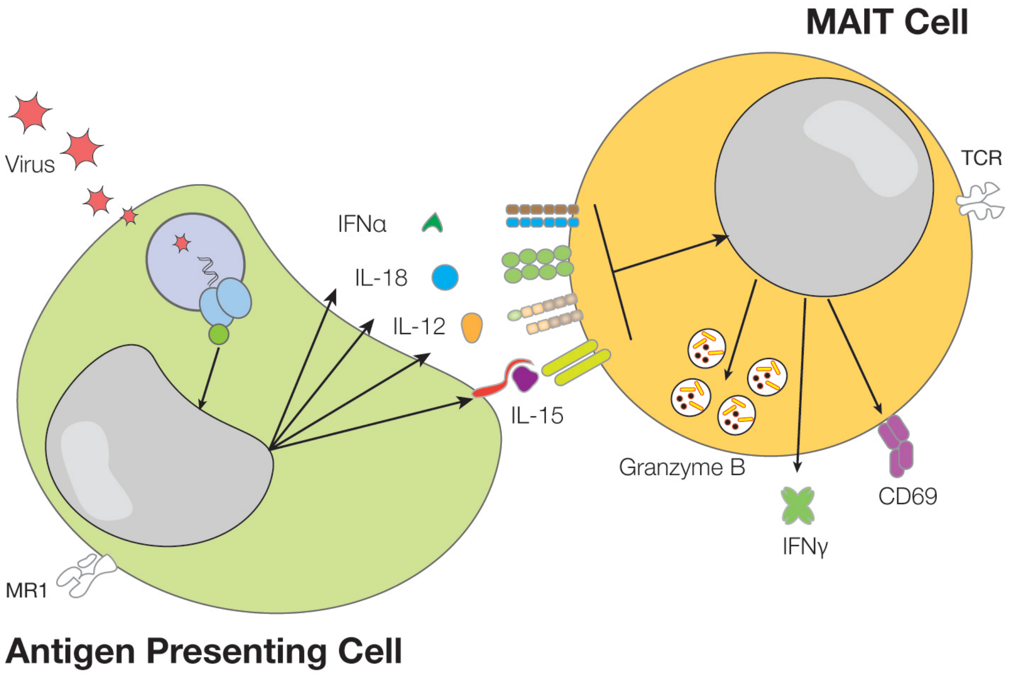

Supplementary Figure 11. Illustrated model of MAIT cell triggering by viruses. In response to viruses, antigen presenting cells, such as Dendritic cells, Alveolar Macrophages and Kupffer cells, are able to activate $CD161^{++}CD8^{+}$ T cells, by expressing cytokines, independent of MR1 antigen presentation. IL-18 in combination with other innate signals, such as IL-12, IL-15 and type I IFNs, is critical in stimulating MAIT cells. MAIT-cells highly express the IL-18 receptor and in response to stimulation express CD69, produce IFN-γ and Granzyme B.

Supplementary Figure 12

a

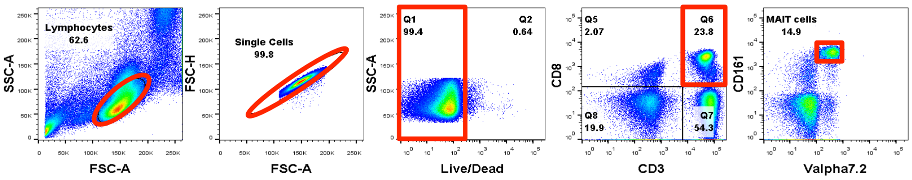

Supplementary Figure 12. Flow cytometry gating strategy used to define MAIT cells.

Supplementary Tables

Supplementary Table 1. Summary of DENV-infected patients enrolled in the study.

Patient #	Sex	JE vac	Age	Diagnosis	Type	Serotype
K01-0029	F	Y	13	DHF2	secondary	DENV1
K01-0023	F	Y	9	DHF1	secondary	DENV1
K01-0030	M	Y	9	DHF1	secondary	DENV2
K02-0032	F	N	11	DHF2	secondary	DENV2
K03-0003	M	unknown	8	DHF3	secondary	DENV1
K03-0005	M	Y	6	DHF2	secondary	DENV1
K02-0018	F	N	9	DHF1	secondary	DENV2
K03-0013	M	Y	10	DHF1	secondary	DENV2
K03-0092	M	N	11	DHF2	secondary	DENV1
K03-0099	M	Y	12	DHF1	secondary	DENV2
K03-0038	M	Y	5	DHF2	secondary	DENV1
K02-0105	F	Y	4	DF	secondary	DENV2
K01-0028	M	unknown	6	DF	secondary	DENV1
K02-0102	F	Y	9	DF	secondary	DENV1
K02-0105	F	Y	4	DF	secondary	DENV2
K02-0016	M	Y	9	DF	secondary	DENV1
K03-0073	F	Y	2	DF	secondary	DENV1
K02-0037	F	Y	12	DF	secondary	DENV1
K03-0063	M	Y	5	DF	secondary	DENV2
K02-0031	M	N	12	DF	secondary	DENV2
K03-0093	M	Y	3	DF	secondary	DENV4
K01-0013	M	unknown	5	DF	primary	DENV1

Clinical characteristics of 20 DENV infected patients suffering from dengue fever (DF) or dengue haemorrhagic fever (DHF1-4). DHF is divided into four grades according to the disease severity: patients with DHF1 and 2 having increased vascular permeability and patients with DHF3 and 4 developing shock due to severe plasma loss. Blood samples were obtained from these individuals at several time points during the acute and convalescent phase of infection. Laboratory confirmation of DENV infection and the serotype (DENV1-4) were determined by RT-PCR detection of DENV nucleic acid. Abbreviations: DENV, dengue virus; DHF, dengue haemorrhagic fever; DF, dengue fever.

Supplementary Table 2. Summary of influenza virus-infected patients.

Patient ID	Sex	Age	Care	Co-morbidities
1	M	48	ICU	
2	F	59	ICU	CLL (since 1992)
3	F	59	ICU	COPD
4	M	54	ICU	COPD
5	F	29	ICU	5 days post partum
6	F	45	ward	Pregnancy (third trimester)
7	F	34	ward	Pregnancy (third trimester)
8	M	60	ward	Diabetes mellitus, epilepsy
9	F	68	ward	CLL

Influenza-infected patients were admitted either to the general ward or ICU. Patients were recruited from two hospitals based in Oxford and Glasgow during the 2009 H1N1 pandemic. Patients had concomitant conditions as documented in Table 2, but were not on immunosuppressant at the point of sampling. Two patients were pregnant, and one was six days post-partum. Abbreviations: CLL, Chronic lymphocytic leukemia; COPD, Chronic obstructive pulmonary disease

Supplementary Table 3. Summary of HCV-infected patients.

treatment naïve

patient ID	gender	age
855	F	44
672	F	36
940	M	32
615	M	41
765	M	52
854	M	36
318	F	49
581	F	60
197	F	53
191	M	64
256	M	55
603	M	52
144	F	47
146	M	56
148	F	68
205/821	M	64
126	M	58

non-response

patient ID	gender	age
191	M	64
262	M	62
306	M	61
327	M	63
349	M	60
383	F	67
391	M	47
403	F	59
594	M	53
500	unknown	unknown
602	M	51
603	M	52
629	F	51
634	M	60
639	F	59
722	f	63

relapse

patient ID	gender	age
120	F	64
144	F	62
146	M	61
148	F	63
152	M	60
185	F	67
226	M	47
279	M	59
307	F	53
376	M	59
205/821	M	53

sustained virological response

patient ID	gender	age
730	M	59
134	F	53
292	M	59
472	M	53
325	M	59
618	M	53
238	M	59
157	M	53
400	M	59
449	M	53
unkown	unkown	
385	M	53

spontaneous clearance

patient ID	gender	age
806	M	41
861	M	55
884	M	39
900	M	48
113	M	67
975	M	53
720	F	64
871	F	62
906	F	61
1014	M	63
1041	M	60
1052	M	67

Samples were collected from the Hepatitis Clinic at the John Radcliffe Hospital, Oxford, UK.

Supplementary Table 4. Summary of HCV-infected patients (BOSON trial).

Patient Number	Treatment	Collection Origin	HCV Genotype
1	SOF + RBV	US	2
2	SOF + RBV	US	3a
3	SOF + RBV	US	3a
4	SOF + RBV	US	2a/2c
5	SOF + RBV	US	3a
6	SOF + RBV	US	3a
7	SOF + RBV	US	3a
8	SOF + RBV	US	3a
9	SOF + RBV	Europe	3a
10	SOF + RBV	Europe	3a
11	SOF + RBV	Europe	3a
12	SOF + RBV	Europe	3c
13	SOF + RBV	Europe	3a
14	SOF + RBV	Europe	3
15	SOF + RBV	Europe	3
16	SOF + PEG/RBV	US	3a
17	SOF + PEG/RBV	US	3a
18	SOF + PEG/RBV	US	3
19	SOF + PEG/RBV	US	3a
20	SOF + PEG/RBV	US	3a
21	SOF + PEG/RBV	US	3a
22	SOF + PEG/RBV	US	3a
23	SOF + PEG/RBV	US	2
24	SOF + PEG/RBV	US	3a
25	SOF + PEG/RBV	US	3b

PBMC from the BOSON clinical trial were used. BOSON is a randomized, open-label, phase 3 study testing Sofosbuvir plus Ribavirin with or without pegylated interferon-alpha in patients with HCV Genotype 3 and Treatment-experienced cirrhotic patients with HCV Genotype 2. PBMCs were processed at one of 3 Covance sites (US, Europe, Singapore).
